# Electrolyte Engineering with Carboranes for Next-Generation Mg Batteries

**DOI:** 10.1021/acscentsci.3c01176

**Published:** 2024-01-12

**Authors:** Anton W. Tomich, Jianjun Chen, Veronica Carta, Juchen Guo, Vincent Lavallo

**Affiliations:** † Department of Chemistry University of California, Riverside, Riverside, California 92521, United States; ‡ Department of Chemical and Environmental Engineering University of California, Riverside, Riverside, California 92521, United States

## Abstract

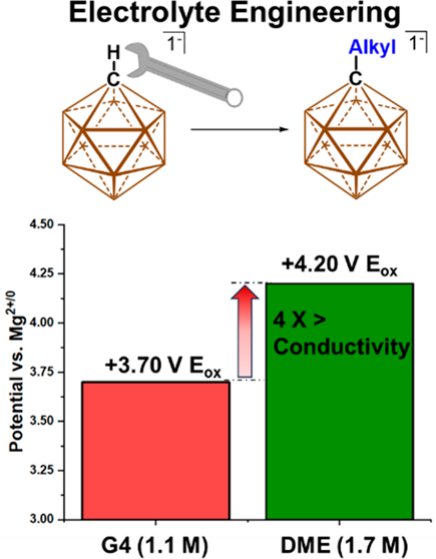

To realize an energy storage transition beyond Li-ion competitive technologies, earth-abundant elements, such as Mg, are needed. Carborane anions are particularly well-suited to realizing magnesium-ion batteries (MIBs), as their inert and weakly coordinating properties beget excellent electrolyte performance. However, utilizing these materials in actual electrochemical cells has been hampered by the reliance on the Mg^2+^ salts of the commercially available [HCB_11_H_11_]^−^ anion, which is not soluble in more weakly binding solvents apart from the higher glymes. Herein, we demonstrate it is possible to iteratively engineer the [HCB_11_H_11_]^−^ anion surface synthetically to address previous solubility issues and yield a highly conductive (up to 7.33 mS cm^–1^) and electrochemically stable (up to +4.2 V vs Mg^2+/0^) magnesium electrolyte that surpasses the state of the art. This novel non-nucleophilic electrolyte exhibits highly dissociative behavior regardless of concentration and is tolerant of prolonged periods of cycling in symmetric cells at high current densities (up to 2.0 mA cm^–2^, 400 h). The hydrocarbon functionalized carborane electrolyte presented here demonstrates >96% Coulombic efficiency when paired with a Mo_6_S_8_ cathode. This approach realizes a needed candidate to discover next-generation cathode materials that can enable the design of practical and commercially viable Mg batteries.

## Abstract 2

A novel strategy to engineer carborane-based magnesium battery electrolytes is reported. The resulting improved solubility, electrochemical stability, and conductivity of the lead material demonstrate the best electrochemical performance to date for Mg batteries.

## Introduction

The demand for improved consumer electronics, widespread adoption of electric vehicles (EVs), and global climate concerns have created overwhelming pressure to develop advanced energy storage technologies. Although these needs have been sated primarily through commercial development of lithium-ion battery (LIB) technologies, a push for cells with even greater energy density has spurred research efforts toward the adoption of pure lithium-metal anodes.^1,2^ These efforts have been severely hindered by the intrinsic properties of lithium, which include its reactivity in the presence of moisture and failure mechanisms that arise from dendritic lithium metal deposits on repeated cell cycling.^3^ Regardless of whether these concerns are alleviated, the rapidly increasing cost of battery-grade Li_2_CO_3_ ($37000 ton^–1^, 2022) and LiOH ($78000 ton^–1^, 2022) and long-term scarcity of lithium raw earth materials will inevitably prohibit the use of LIBs as a sustainable means of energy storage.^4,5^ Thus, alternative electrochemical systems that relieve these concerns while improving upon current energy density metrics are essential to realizing the energy transition away from fossil fuels.

An exceedingly attractive alternative to lithium are electrochemical systems based on magnesium, whose high crustal abundance (Mg 2% vs Li 0.002%) stands to mitigate many of the concerns associated with raw material cost and scarcity.^6^ Pure magnesium-metal anodes exhibit a volumetric capacity that nearly doubles that of lithium due to its divalent charge (3833 mAh cm^–3^ vs 2046 mAh cm^–3^ Li) and low reduction potential amenable to high-voltage batteries (−2.37 V vs SHE).^7,8^ Additionally, magnesium metal lends to an inherently safer cell chemistry, as it is significantly less pyrophoric and tends not to form dendritic metal morphologies over extended periods of cell cycling.^9,10^ These traits have made commercial magnesium-ion batteries (MIBs) very desirable; however, their realization is not without its own challenges. Most electrolyte components commonly employed in LIBs exhibit fatal incompatibilities with magnesium metal anodes due to their chemical and electrochemical instability which results in Mg^2+^ insulating passivation films to form on the anode surface.^11,12^ For this reason, magnesium electrolytes that exhibit absolute stability under the reducing conditions of Mg metal have long been recognized as being necessary to mediate reversible behavior in these systems. To date, a number of successful novel liquid electrolytes, polymer electrolytes, additives, and ex situ surface engineering techniques have been employed to prolong cell cycling in magnesium-based systems.^13−15^ However, even prominent candidates impose limitations, including low conductivity, nucleophilicity, and chemical instability.

**Figure 1:**
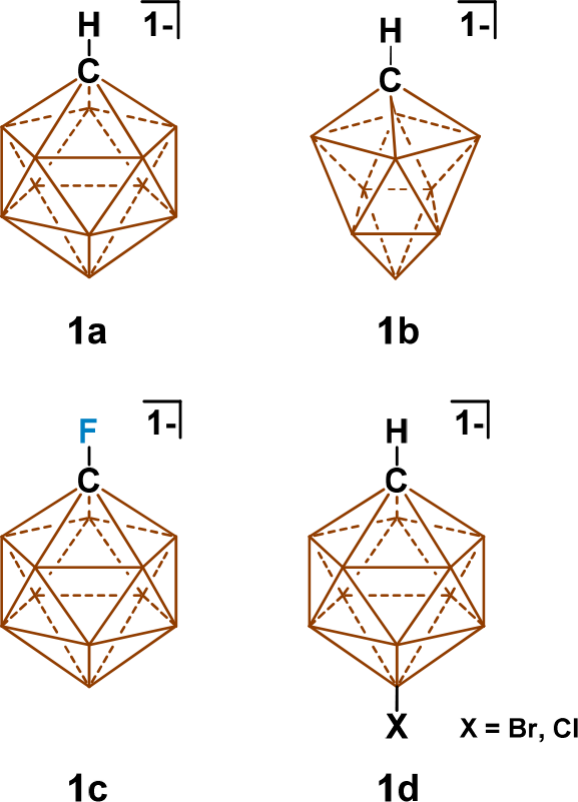
Carborane anions previously employed in magnesium electrolytes.

In molecular chemistry, the icosahedral carborane anion [HCB_11_H_11_]^−^ (**1a**) and its polyhalogenated derivatives are distinguished among weakly coordinating anions (WCAs), as they have no equals with respect to the aggregate properties of thermal/chemical stability and weakly coordinating ability.^16−22^ Additionally, the electrochemical stability of the **1a** anion has no comparable counterpart in other families of weakly coordinating anions when considering both the oxidative and reductive ends of the spectrum (+6.0 V oxidative stability and immune to Li-metal reduction).^16,23^ The reason this molecule is bestowed with these properties is it has very delocalized chemical bonding and an enormous HOMO–LUMO gap.^24^ As a consequence **1a**, and its smaller counterpart **1b**, are inherently noncorrosive and nontoxic and are not known to decompose into deleterious byproducts. While halogenated carboranes are more weakly coordinating and thought to impart improved ionic conductivity to the electrolyte, they are not universally compatible with alkali-metal anodes, as the B–X or C–X (X = F, Cl, Br, I) bonds are susceptible to reduction.^16^ Previously, our group and that of Mohtadi et al. concurrently and independently developed the salt [Mg^2+^][HCB_11_H_11_]^−^_2_ (Mg**1a**) as an electrolyte component which enables exceptional Mg battery performance compared to all other known electrolytes.^25−27^ Although **1a** has been established as a reliable anionic component, prior explored formulations display lackluster conductive properties and oxidative stability due to the solubility properties of Mg**1a**, which constrains electrolyte formulations to higher glymes. The full potential of Mg**1a** cannot be realized, as its properties are limited by the electrochemical stability, polydenticity, and viscous nature of these solvents. In one iteration of a carborane electrolyte previously investigated by our group, we addressed these grievances via a Grignard species, **1a**MgPh, which exhibited extraordinarily high conductivity and oxidative stability.^28^ However, this strategy introduced a degree of nucleophilicity and moisture sensitivity to the electrolyte, which precluded its further adoption. A distinct, underexplored advantage to subvert these issues is through direct modification of the anion, which has previously been the subject of fundamental investigations with carborane derivatives.^29^ One such report describes an increase in anion stability with Mg**1c** to +4.9 V vs Mg^2+/0^, a limit that is accompanied by the formation of solvent adducts (Figure 1).^30^ However (vide supra) anions such as **1c**,**d** are unsuitable for these systems as C–X and B–X bonds may be vulnerable to reduction by alkali metals, which introduces passivating species to the interphase and liberates molecular halogens that are corrosive to cell casings (Figure 1). While this study, among others, provides a crucial understanding of anion properties and stability limits, practical improvements over carboranyl electrolytes as well as their incorporation in MIBs are not presented. To date, there are over 300 known functional derivatives of **1a**,**b**, few of which have been adequately explored as MIB electrolyte candidates.^21^

Herein we report that hydrocarbon functionalities can be molecularly engineered via covalent linkages to the carbon vertex without a loss of stability under the reported operating conditions. Additionally, the optimized formulation of these alkylated carborane electrolytes possesses conductivity, oxidative stability, and solubility properties that are significantly improved over those of Mg**1a** electrolytes. The inert alkyl functionality yields a carboranyl magnesium salt whose improved DME solubility enables 4× greater conductivity than previous formulations of Mg**1a**, and an oxidative stability of +4.2 V vs Mg^2+/0^. The highly conductive electrolyte exhibits low polarization in symmetrical Mg–Mg cells cycled at high current densities (2.0 mA cm^–2^) beyond 400 h, and Mg||Mo_6_S_8_ cells assembled with the electrolyte exhibit a Coulombic efficiency of >96%. For the first time, spectroscopic investigations of carboranyl magnesium electrolytes confirm that the in situ solvation structure is effectively decoupled from electrolyte concentration due to the anion’s weakly coordinating properties. Importantly, we demonstrate that the synthetic modularity of **1a** is a distinct advantage over traditional WCAs to tune electrolyte performance.

## Results and Discussion

**Figure 2:**
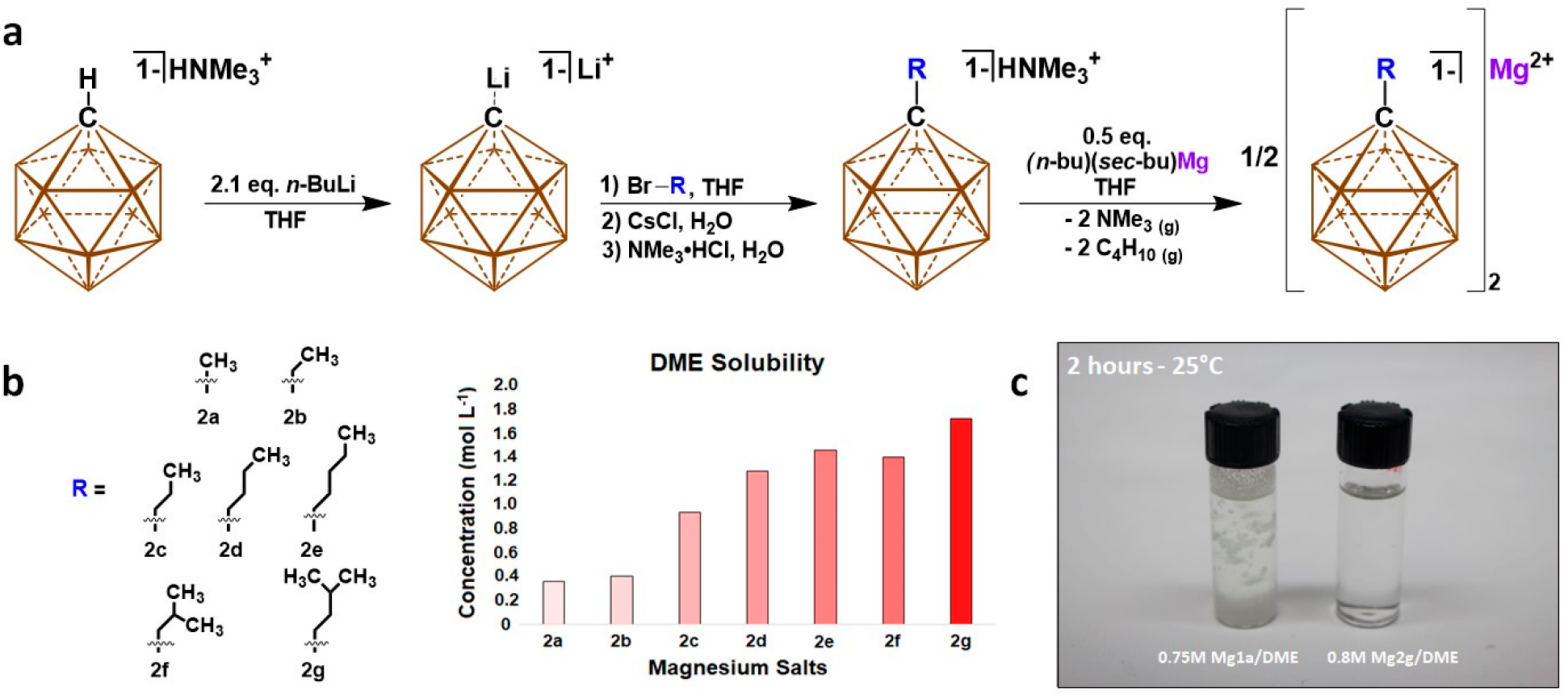
(a) Synthetic pathway toward a series of alkylated carboranyl magnesium salts. (b) Graph displaying DME solubility of alkylated anion Mg salts as a function of chain length and branching. (c) Digital image of concentrated Mg salt/DME solutions allowed to warm for 1 h from −30 °C: (left) Mg**1a** in DME and (right) Mg**2g** in DME.

During the course of a previous investigation, we identified a branched alkyl moiety appended at the C-vertex of the carborane anion that prevented the gradual crystallization of a carboranyl silver salt in a eutectic ionic liquid solution.^31^ For the purpose of addressing the solubility issues of Mg**1a** and pairing with magnesium metal anodes, a similar modification would be ideal, as the hydrocarbon moiety should impart relatively inert chemical and electrochemical influences on the anion. Functionalized anions and magnesium salts were prepared from modified known procedures to yield a small library of alkylated carborane species that could be prepared rapidly in high yield via reaction of corresponding trimethylammonium salts with *n*-butyl *sec*-butyl magnesium (Figure 2a). This method yields magnesium salts of high purity, as all salt exchange biproducts are gaseous and effervesce from the solution as the reaction progresses. All species were characterized by multinuclear NMR and HR-MS to verify their successful functionalization and purity (Figures S1–S35). Upon alkylation, a distinct shift occurs in the ^11^B{^1^H} NMR spectra, which assists in ensuring the purity of the resulting salts (Figure S36). We were initially disappointed to find alkylated species Mg**2a** and Mg**2b** afford little more DME solubility than Mg**1a** (Figure 2b). In fact, Mg**2a** displays the same elevated temperature crystallization behavior as Mg**1a** in DME (Figure S37). Gratifyingly, the DME solubility of the Mg**2** series increased significantly as a function of the alkyl chain length and afforded a means to effectively tune the solubility properties of the magnesium species. A maximum solubility of 1.72 M was observed for species Mg**2g**, which we attribute to the increased van der Waals interactions afforded by the isopentyl moiety (Figure 2b). It was noted that functionalized carborane anions wielding an alkyl moiety of 3 or fewer carbons did not impart sufficient solubility for the preparation of concentrated electrolyte solutions, and generally a lack of branching in alkylated species tended to encourage gradual crystallization of the Mg**2a**–**e** species over time. Moreover, saturated DME solutions of all Mg**2a**–**g** species tended to crystallize slowly, precluding their use at solubility limits (Figure S38). In anticipation of studying the conductive properties and in situ solvation structure of a Mg**2** species over a wide concentration range, anion **2g** was upselected as the preferred material for study on the basis of the high solubility afforded in DME and lack of crystallization behavior. Indeed, 1.4 M DME solutions of Mg**2g** exhibit no crystallization at or above room temperature (Figure 2c and Figure S39).

**Figure 3:**
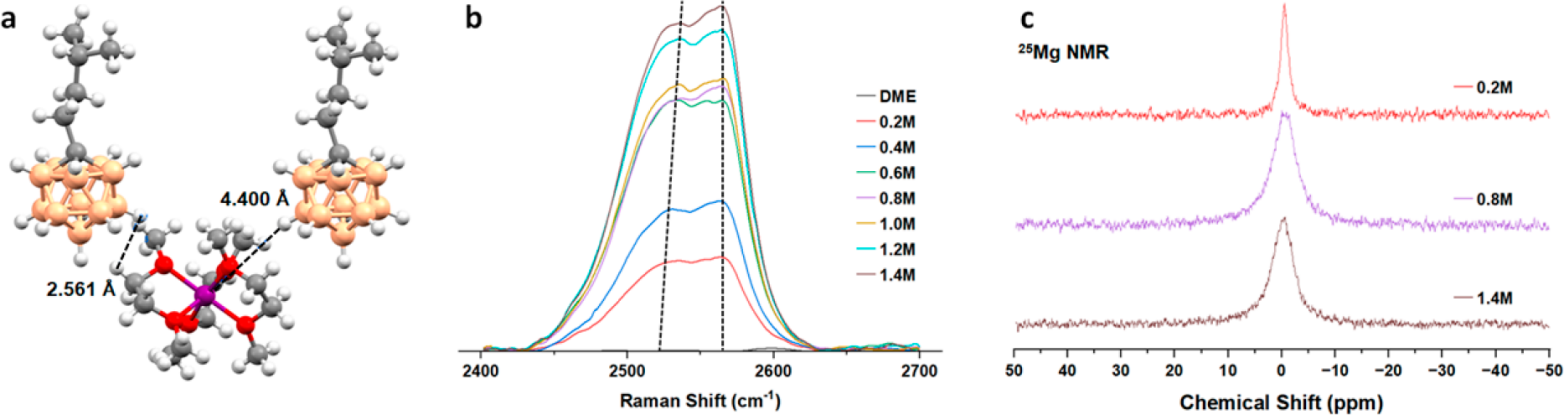
(a) Single-crystal X-ray structure of Mg**2g** in the solid state. (b) Raman shift of anion **2g** B–H as a function of concentration in the 2400–2700 cm^–1^ region. (c) ^25^Mg NMR spectra of Mg**2g** in DME at dilute (red), moderate (purple), and high (brown) concentrations.

Mg**2g** was characterized via multinuclear NMR and Raman spectroscopy as a means to evaluate the material purity and offer insight into the in situ solvation structure of functionalized carboranyl electrolytes. The concentration-dependent solvation structure of carboranyl magnesium electrolytes has not been reported previously, though we suspected the highly delocalized charge would preclude the formation of contact ion pairs in situ, aid in ion dissociation, and yield improved electrolyte conductivity. A crystal structure of Mg**2g** in the solid state was obtained which corroborates the multinuclear NMR data and portrays a single Mg^2+^ cation solvated by 3 equiv of DME charge balanced by two monoanionic carboranes (Figure 3a). The closest interaction of the Mg^2+^ cation with the B–H of anion **2g** was found to be 4.4 Å, which is well outside the range of any significant bonding interaction, yielding Mg**2g** as a solvent-separated ion pair (SSIP). The cluster B–H bonds exhibit interaction distances with solvating DME between 2.55 and 2.70 Å, which suggests some degree of hydrogen bonding occurs between the anion and solvation shell. The solubility of this species was employed as a means to explore the in situ solvation structure of the electrolyte over a range of concentrations (0.2–1.4 M) in DME via Raman and ^25^ Mg NMR spectroscopy. In the high-frequency region (2400–2700 cm^–1^) anion **2g** displays a distinct stretching frequency corresponding to cluster B–H vertices which may interact strongly with Mg^2+^ at high salt concentrations._._ It was anticipated that strong interactions would result in shifted B–H signals indicative of contact-ion pair formation which significantly influences cation diffusion and ionic conductivity.^100,101^ Instead, a small Raman shift of ∼6 cm^–1^ is observed over the entire investigated range, suggesting there is little difference in the degree of the B–H–Mg^2+^ interaction, which we ascribe to increased SSIP speciation at higher concentration (Figure 3b). No Raman shift is observed for the peak at 2565 cm^–1^ over the range of concentrations investigated here, which allows us to tentatively assign it to B–H comprising the upper pentagonal belt of the cluster. Likewise, no Raman shift is detected in peaks unique to the carborane anion in the low-frequency range (700–900 cm^–1^). A peak at 881 cm^–1^ is observed to grow with increasing concentration of Mg**2g**, which has previously been identified as a bending mode of DME in the [Mg(DME)_3_]^2+^ complex (Figure S40).^32^ This peak is observable at even low concentrations and becomes more prominent with increasing concentration, suggesting that the solvation of Mg^2+^ and formation of the SSIP species are highly favorable. ^25^Mg NMR was employed as a means to characterize the chemical environment of Mg^2+^ and corroborate results observed in the Raman spectra. Dilute and near-saturation DME solutions of Mg**2g** exhibited a chemical shift of −0.33 ppm, which is well within the expected range for a free Mg^2+^ cation (Figure 3c). Increasing salt concentration begets a broadened Mg^2+^ peak arising from interactions of the coordinatively saturated cation with B–H which we correlate to an increased population of SSIP species not present at very low concentrations.^24^ Here, we demonstrate formation of contact-ion pairs or aggregate complexes is decoupled from the electrolyte concentration, and the SSIP species remain predominant in the Mg**2g**/DME electrolyte. This observation speaks to the highly weakly coordinating nature maintained by anion **2g** and gives credence to recent computational works detailing ion speciation in a glyme-based electrolyte based on anion **1a**.^24^

**Figure 4:**
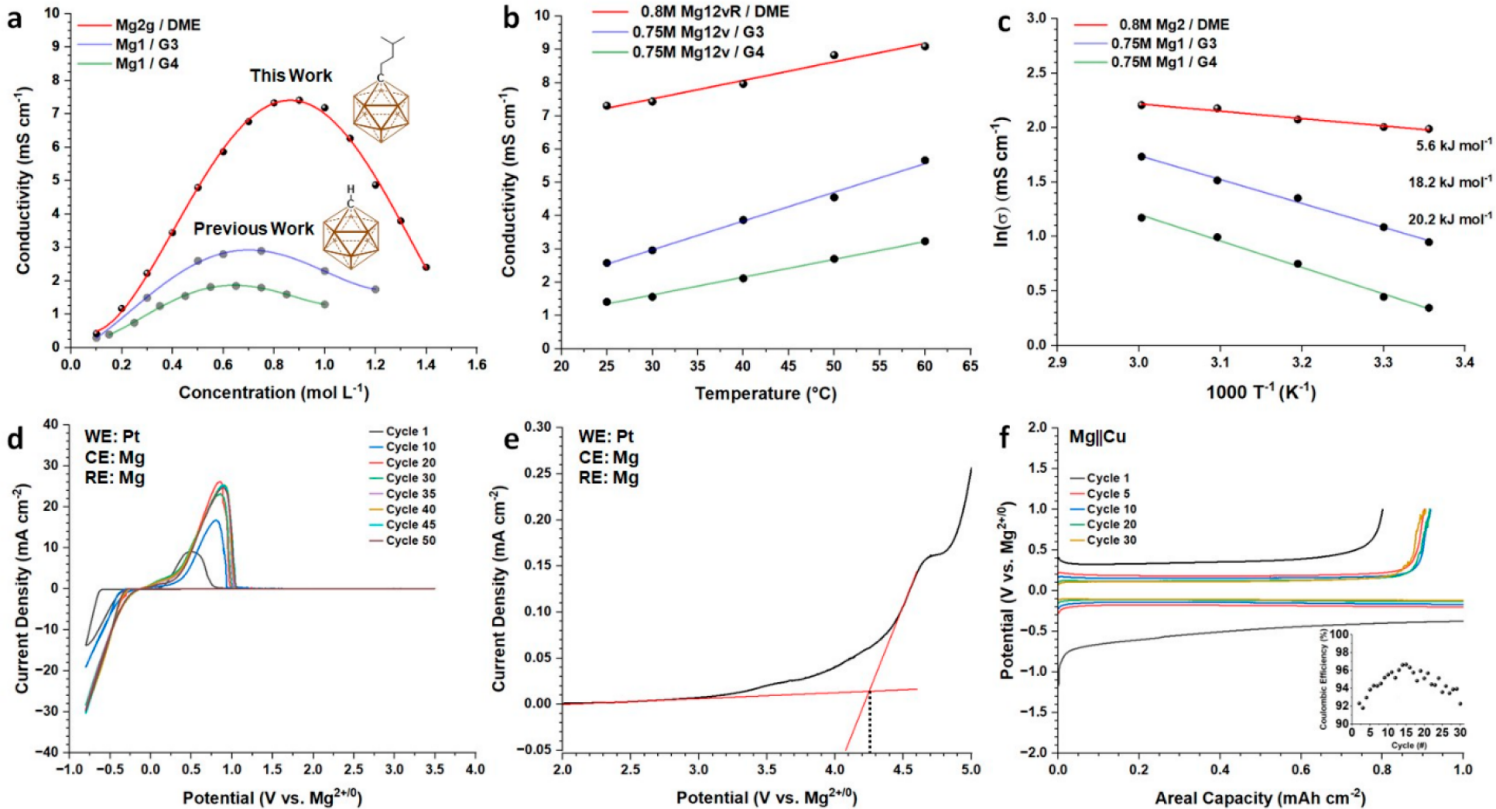
Electrochemical characterization of Mg**2g**/DME. (a) Conductivity as a function of concentration for Mg**2g**/DME solutions overlaid with previously reported formulations (reproduced from ref (11)). (b) Conductivity as a function of temperature for Mg**2g**/DME and previously reported Mg**1a** electrolytes. (c) Arrhenius plot of Mg**2g**/DME and previously reported Mg**1** electrolytes with calculated *E*_a_. (d) 3-electrode cyclic voltammetry of Mg**2g**/DME on Pt from −0.8 to 3.5 V vs Mg^2+/0^ (cycles 1–50, 10 mV s^–1^). (e) Anodic sweep of Mg**2g**/DME on Pt (5 mV s^–1^). (f) Mg||Cu constant current deposition (inset: % Coulombic efficiency).

Electrochemical impedance spectroscopy (EIS) was employed to measure the conductive properties of Mg**2g** in DME to determine the optimal electrolyte formulation. To this end, the conductivity was measured over the range of concentration afforded by Mg**2g**. A maximum conductivity of 7.4 mS cm^–1^ was observed for the 0.9 M Mg**2g** DME formulation, which constitutes a nearly 4× improvement over the previously preferred formulation of Mg**1**/G4 (Figure 4a). Little variation was observed over the concentration range 0.8–1.0 M for Mg**2g** DME solutions, and ionic conductivity between 0.8 and 0.9 M solutions constituted a difference of ±0.07 mS cm^–1^. For this investigation, 0.8 M Mg**2g** in DME (Mg**2g**/DME) was chosen as the preferred candidate for further study, in favor of preserving the quantity of conducting salt used in electrolyte formulation. It was noted that the conductivity of 0.8 M Mg**2g** in G4 (σ = 1.1 mS cm^–1^) is lower than the conductivity reported for the 0.75 M Mg**1a**/G4 electrolyte (σ = 1.8 mS cm^–1^) and substantially lower than that of Mg**2g**/DME (7.33 mS cm^–1^) (Figure S41). This alleviates the notion that the functionalized anion **2g** is a contributor to the higher observed conductivity and affirms that the reduced viscosity afforded by DME is the defining factor. In fact, the lower conductivity observed in G4 solutions may arise from increased intermolecular interactions via the anions’ isopentyl moiety. The elevated temperature conductivity of both Mg**2g**/DME and prior unfunctionalized formulations were investigated as a means of detailing electrolyte transport behavior and observing electrolyte conductivity at practical device operating temperatures. A maximum conductivity of 9.1 mS cm^–1^ was observed for Mg**2g**/DME at 60 °C relative to the 3.2 mS cm^–1^ observed for Mg**1a**/G4 at the same temperature, demonstrating that even slightly elevated temperatures yield behavior approaching 10^–2^ S cm^–1^ for the novel formulation (Figure 4b and Figure S42). The room-temperature and elevated-temperature conductivities both represent a significant improvement over a handful of commonly employed MIB electrolytes, including TFSI^–^ and some Al(HFIP)_4_^–^ formulations (Figure S43). The carboranyl electrolytes investigated here exhibit classic Arrhenius behavior, and the activation energies of ion diffusion (*E*_a_) for Mg**1a**/G4 (20.2 kJ mol^–1^) and Mg**1a**/G3 (18.2 kJ mol^–1^) were found to be of similar magnitude to those of many magnesium ionic liquid and polymer electrolytes. In contrast, the *E*_a_ for Mg**2g**/DME (5.6 kJ mol^–1^) is almost 4× less than that of the Mg**1a**/G4 formulation, which adequately explains the large observed differences in conductivity that arise from using a significantly less viscous glyme (Figure 4c).

Further electrochemical characterization of Mg**2g**/DME was undertaken to evaluate the reversible plating and stripping behavior of the magnesium metal. Mg**2g**/DME exhibits a first-cycle overpotential of 600 mV which quickly decreases to 300 mV upon conditioning of the electrode surface after the first 10 cycles (Figure 4d). After 25 cycles, the overpotential associated with deposition and dissolution of magnesium decreased to 260 mV, highlighting the favorable interfacial chemistry between Mg**2g**/DME and the electrode surface (Figure S44). A high current density is sustained in these processes over 50 cycles owing to the highly reversible behavior relied upon by carboranyl electrolytes. Subjecting Mg**2g**/DME to highly oxidizing potentials reveals the exceptional stability of the electrolyte (+4.2 V vs Mg^2+/0^), which represents a significant expansion of the electrochemical window over the unfunctionalized Mg**1a**/G4 electrolyte (+3.7 V vs Mg^2+/0^) (Figure 4e). Rigorous characterization of the reversible behavior of Mg**2g** was undertaken through constant current deposition/dissolution of magnesium metal from a copper substrate. Mg**2g**/DME displayed a voltage hysteresis which decreased to <200 mV and an average Coulombic efficiency of 94.2% over the course of cycling (Figure 4f). This suite of data satisfies curiosity regarding electrolytes utilizing **1a**, whose anodic stability appears to be solvent limited rather than derived from anion oxidation. A prior report from our group of a Grignard species employing **1a** was shown to exhibit an oxidative stability of up to +4.6 V vs Mg^2+/0^, which is congruent with investigations into the absolute anodic stability of **1a**.^28,30^ Thus, the increased anodic stability enjoyed by Mg**2g**/DME is a consequence of the favorable solubility properties afforded by the anion, while also being absent the nucleophilic and moisture-sensitive nature of the Grignard species. Few magnesium electrolytes have been reported with an electrochemical window that extends beyond +4.0 V vs Mg^2+/0^, and Mg**2g**/DME demonstrates that the highly modular carborane anion is a promising platform toward high-voltage MIBs.^33,34^

**Figure 5:**
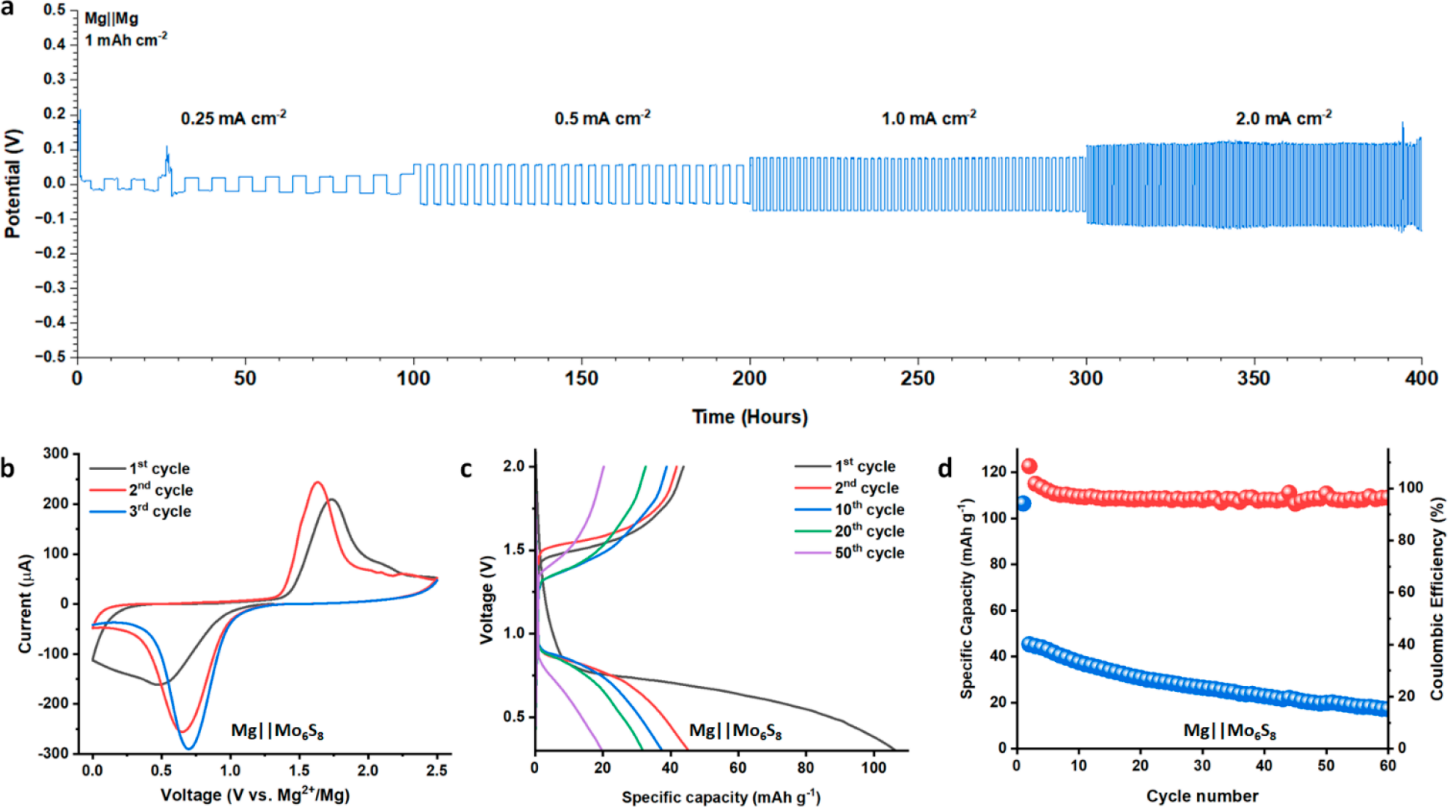
(a) Symmetrical Mg||Mg cells cycled containing Mg**2g**/DME at 1 mAh cm^–2^ areal capacity over increasing current density. (b) Cyclic voltammograms of Mg||Mo_6_S_8_ cell containing Mg**2g**/DME (0.1 mV s^–1^). (c) Voltage profile of Mg||Mo_6_S_8_ half cell containing Mg**2g**/DME at 0.1 C over 50 cycles. (d) Cycling performance of Mg||Mo_6_S_8_ cell with Mg**2g**/DME at 0.1 C.

Symmetrical Mg–Mg cells were assembled containing Mg**2g**/DME to evaluate the chemical and electrochemical compatibility of anion **2g** with magnesium metal as well as the reversible behavior of Mg**2g**/DME over prolonged cycling. Surprisingly, Mg–Mg cells containing Mg**2g**/DME exhibit a very low overpotential of 58.1 mV when subjected to a current density of 0.5 mA cm^–2^ over 400 h of cycling (Figure 5a and Figure S45). Similarly low cell polarization was observed at high current densities, with average current responses of 86 and 106.6 mV for 1.0 and 2.0 mA cm^–2^ cells, respectively (Figures S46 and S47). The remarkably low cell polarization observed here may be attributed to the low internal cell resistance afforded by Mg**2g**/DME and the inert nature of **2g** that yields an anode surface free of anion reduction products. Contemporary electrolyte design has recognized that anions with high fluorine content may contribute to a MgF_2_-rich SEI which enables unusually stable deep cycling of magnesium metal anode cells.^102^ Given that there is no fluorine or fluoride-containing reagents involved in the synthesis or preparation of Mg**2g**/DME, halogen-free anions **1a** and **2g** suggest this design principle may not be necessary to achieve prolonged cycling behavior.

The reversible behavior of Mg**2g**/DME was further investigated in Mg||Cu and Mg||Mo_6_S_8_ half-cell configurations. Mg||Cu half-cells were assembled containing Mg**2g**/DME in an effort to correlate the favorable symmetrical cell performance to the magnesium metal deposition morphology. Scanning electron microscopy (SEM) paired with energy dispersive X-ray spectroscopy (EDS) mapping reveals flat, dense magnesium metal deposition on the copper substrate free of dendritic metal morphologies (Figure S48). Characterization of the deposited magnesium metal surface also reveals a distinct lack of boron-containing species indicative of the maintained integrity of anion **2g** (Figure S49). The electrolyte chemical stability in the reducing environment is further highlighted by the high Coulombic efficiency associated with the reversible deposition/dissolution of magnesium (Figure 4f and Figure S50). Lastly, Mg||Mo_6_S_8_ cells were assembled containing Mg**2g**/DME to characterize the behavior of the electrolyte paired with a suitable cathode material. Two-electrode cyclic voltammetry of the half-cells operated at 25 °C correlates well with the discharge–charge behavior (Figure 5b). A first-cycle discharge capacity of 125 mAh g^–1^ was obtained when the cell was cycled at 0.01 C, corresponding to a 97% theoretical capacity utilization of the Mo_6_S_8_ cathode (Figure S51a). This behavior was not reversible due to charge transfer limitations between Mg^2+^ and the electrode, and significant capacity fade was observed over time. Similar performance was observed in cells cycled at 0.1 C, which were capable of maintaining an average 34 mAh g^–1^ capacity over the first 30 cycles (Figure 5c,d). Previously reported carboranyl electrolytes paired with Mo_6_S_8_ displayed similar behavior, although they were not capable of the high initial discharge capacity exhibited by cells containing Mg**2g**/DME.^27^ A greater reversible capacity (∼60 mAh g^–1^) was obtained when the cells were cycled at slightly elevated temperatures; however, they suffered from poor cycling stability (Figure S51b). Notably the half-cell performance with Mg**2g**/DME is facilitated by a >96% CE, which bodes well for future cathode pairing pursuits.

## Conclusion

Current approaches to magnesium electrolyte development are typically constrained to the introduction of new solvent or salt additives, concentration-dependent performance characteristics, or in situ electrolyte speciation derived from multiple chemical reagents. Although these practices are an effective means to improved electrolyte performance, their breadth is often limited in comparison to the targeted synthesis of novel anions and simple salts.^35,36^ The molecular engineering of alkyl groups onto the C-vertex of salt **1a** represents a novel paradigm in electrolyte engineering, which avoids the prior efforts of others that incorporate reductively unstable halogens onto the cluster surface. Here, we have developed a novel liquid electrolyte that boasts improved solubility over a wide temperature range, a high ionic conductivity (7.33 mS cm^–1^), and an increased oxidative stability by +0.5 V (+4.2 V vs Mg^2+/0^) in comparison to prior formulations hindered by viscous glymes. Our investigations reveal a previously unexplored aspect of carboranyl magnesium electrolytes regarding their propensity for SSIPs to remain the dominant species in situ, which likely contributes to their favorable transport properties. Lastly, we demonstrate highly reversible symmetrical and half-cell configurations utilizing Mg**2g**/DME, which bodes well for future attempts to pair the electrolyte with high-voltage cathodes. Essential to this work is the maintenance of favorable properties of **1a** that are not influenced by chemical functionalization, including the chemical stability and weakly coordinating behavior of the anion. Future functionalized derivatives of **1a**, or any BRN species, should take care to adopt similar design principles so that performance benefits derived from these species are preserved. Given the plethora of synthetic methodologies available for the chemical functionalization of anion **1a**, many functionalized carborane anions exist, or can be synthetically modified, that may impart exciting new properties upon magnesium and other alkali- or alkali-earth-metal electrolytes. Moreover, simple, scalable, and cost-effective access to **1a** has been realized, poising carboranyl electrolytes as an economically viable alternative to WCAs commonly employed in energy storage technologies.^37,38^ Most importantly, having these new materials and the modular approach described above in a multidisciplinary battery research team’s arsenal will allow for the identification of novel high-voltage and -capacity cathode materials for Mg batteries and beyond that will facilitate global electrification.

## Supporting Information Available

Crystallographic data for Mg**2g** (CIF)
Experimental procedures, detailed synthetic procedures and characterization (multinuclear NMR, HR-MS, Raman spectroscopy), digital images of crystallization behavior, literature produced conductivity table, detailed electrochemical characterization, surface characterization, and single-crystal data (PDF)

## Supplementary Material

oc3c01176_si_001.cif
