# Supplementary material for: Electrolyte Engineering with Carboranes for Next-Generation Mg Batteries
Source: ACS Cent Sci. 2024 Jan 12;10(2):264–71. doi: 10.1021/acscentsci.3c01176 (PMC10906036; doi:10.1021/acscentsci.3c01176)
Supplement: Supplementary file 2 — oc3c01176_si_002.pdf [file oc3c01176_si_002.pdf]

Electrolyte Engineering with Carboranes for Next Generation Mg-Batteries

Dr. Anton W. Tomich,[a] Jianjun Chen,[b] Dr. Veronica Carta,[a] Dr. Juchen Guo,[b]* and Dr. Vincent Lavallo[a]*

[a] Department of Chemistry, University of California Riverside, Riverside, CA 92521, United States

[b] Department of Chemical and Environmental Engineering, University of California Riverside, Riverside, CA 92521, United States

Email: vincent.lavallo@ucr.edu, jguo@engr.ucr.edu

This supporting document includes :

Material Synthesis and Experimental Methods

Figures S1 – 52

Table S1

References

Table of Contents

Experimental Procedures **3**
Spectroscopic Characterization **6**
Mass Spectrometry **20**
Electrolyte Crystallization Behavior **24**
Raman Spectroscopy **25**
Electrochemistry & Surface Characterization **26**
Single Crystal Data – Mg2g **32**

Experimental Procedures

General Considerations

All manipulations were carried out using standard Schlenk or glovebox techniques under a dinitrogen or UHP (99.995%) argon atmosphere (glovebox, Schlenk line) unless otherwise stated. Dry THF and DME was obtained via distillation under argon from potassium using benzophenone ketyl radical as an indicator. Unless specifically stated, reagents were purchased from commercial vendors and used without further purification. Nuclear magnetic resonance (NMR) spectroscopy was carried out using: Bruker Avance 600 MHz and Bruker NEO 400 MHz (Prodigy LN2 cryoprobe), Varian Inova 500 MHz and Bruker NEO 600 (CP-MAS). NMR chemical shifts are reported in parts per million (ppm) with ^{1}H and ^{13}C chemical shifts referenced to the residual non-deutero solvent. High-resolution mass spectrometry (HRMS) was collected on an Agilent Technologies 6210 (TOF LC/MS) featuring a direct injection with multimode electrospray ionization/atmospheric-pressure chemical ionization (ESI/APCI).

Synthesis of Anion Series 2

$Cs^+[HCB_{11}H_{11}]^-$ was prepared following known literature procedures from decaborane ($B_{10}H_{14}$) which was sublimed prior to use.[1] $[HNMe_3]^+[HCB_{11}H_{11}]^-$ was prepared by dissolution of $Cs^+[HCB_{11}H_{11}]^-$ in warm water followed by the addition of $NMe_3{\cdot}HCl$, and the solid was collected via vacuum filtration. $[HNMe_3]^+[HCB_{11}H_{11}]^-$ was dried for 12 hours in vacuo at 170°C. Alkylated carborane anions were prepared from a modified literature procedure[2]: Working in a glovebox, $[HNMe_3]^+[HCB_{11}H_{11}]^-$ was dissolved in THF and 2.5 eq. of *n*-BuLi (2.5 M solution in hexane) was added dropwise following concentration under vacuum. Once the effervescence of NMe_3 and butane gas seized, the reaction was allowed to stir for 1 hour before confirming complete conversion to dianionic species $[CB11H11]^{2-}[Li]^{+}_{2}$ by $^{11}B\{^{1}H\}$ NMR. The THF solution containing dianionic species $[CB11H11]^{2-}[Li]^{+}_{2}$ was added dropwise to a stirring solution of pentane or hexane approximately 2/1 v/v the volume of the crude reaction mixture during which a white precipitate formed. The precipitate was allowed to settle and the pentane/hexane were carefully decanted. Remaining pentane/hexane was removed under vacuum. To the solid, 1.1 mol eq. of alkyl bromide were added followed by a minimum of THF. The reaction was typically complete after 3 hours, but was often left to stir overnight. The crude reaction mixture was precipitated in hexanes followed by dissolution in a minimum of water yielding a stirring biphasic solution of $[R\text{-}CB_{11}H_{11}]^-[Li]^+$. CsCl was added and the solution was allowed to stir for 15 minutes as $[R\text{-}CB_{11}H_{11}]^-[Cs]^+$ gradually precipitated. The slurry was filtered and allowed to dry yielding crude $[R\text{-}CB_{11}H_{11}]^-[Cs]^+$ as a white solid. $[R\text{-}CB_{11}H_{11}]^-[Cs]^+$ was crystallized from water to yield a white, pearlescent material.

Synthesis of Magnesium Salts

$[R\text{-}CB_{11}H_{11}]^-[Cs]^+$ was stirred on equal v/v solution of water/$CHCl_3$ to obtain a slurry. 1.5 mol eq. NMe_3HCl was added to the slurry and the mixture was stirred vigorously for 15 minutes. The biphasic solution was allowed to separate into two layers, and the $CHCl_3$ containing $[R\text{-}CB_{11}H_{11}]^-[HNMe_3]^+$ was separated and dried in-vacuo to yield $[R\text{-}CB_{11}H_{11}]^-[HNMe_3]^+$ as a white powder. This powder was stirred in a minimum of cold water for 15 minutes, filtered (2x), and allowed to dry. The solid was collected and dried *in-vacuo* at 80 °C for a minimum of 24 hours. Working in a glovebox, the dry $[R\text{-}CB_{11}H_{11}]^-[HNMe_3]^+$ was dissolved in a minimum of THF and 0.65 mol eq. *n*-Bu *sec*-Bu magnesium was added dropwise to the stirring solution upon which $([R\text{-}CB_{11}H_{11}]^-)_2Mg^{2+}$ immediately precipitated. The reaction was allowed to stir for 1 hour upon which 40 mL of hexane was added, and the solid $([R\text{-}CB_{11}H_{11}]^-)_2[Mg(THF)_6]^{2+}$ was collected via vacuum filtration. The $([R\text{-}CB_{11}H_{11}]^-)_2[Mg(THF)_6]^{2+}$ species were recrystallized by THF/Hexane vapor diffusion from a minimum of THF in which the species exhibit limited solubility. The crystalline, white solid was dissolved in 10 mL of dimethoxyethane (DME), stirred for 30 minutes, and dried *in-vacuo* to yield $([R\text{-}CB_{11}H_{11}]^-)_2[Mg(DME)_3]^{2+}$ in high purity.

Mg**2a**: $^{11}B\{^{1}H\}$ NMR (128 MHz, D_6-Acetone): ∂ = -10.06 ppm (bs, 1H, B-H), -11.83 ppm (bs, 10H, B-H). ^{11}B NMR (128 MHz, D_6-Acetone): ∂ = 9.71, 10.42, 11.44, 12.19 ppm. ^{1}H NMR (400 MHz, D_6-Acetone): ∂ = 3.48 (s, 12H), 3.29 (s, 18H) 1.50 (s, 6H) ppm. ^{13}C NMR (100 MHz, D_6-acetone): ∂ = 71.49, 64.37, 58.02, 27.09 ppm. HRMS $\{CH_3CB_{11}H_{11}^-\}$ = calculated: 157.21917 m/z, found: 157.2214 m/z.

Mg**2b**: $^{11}B\{^{1}H\}$ NMR (128 MHz, D_6-Acetone): ∂ = -7.50 ppm (bs, 1H, B-H), -11.09 ppm (bs, 10H, B-H). ^{11}B NMR (128 MHz, D_6-Acetone): ∂ = -6.98, -8.04, -10.54, -11.73 ppm. ^{1}H NMR (400 MHz, D_6-Acetone): ∂ = 3.47 (s, 12H), 3.29 (s, 18H), 1.85 (q, 4H), 0.85 (t, 6H) ppm. ^{13}C NMR (100 MHz, D_6-acetone): ∂ = 72.34, 71.64, 58.75, 33.08, 14.90 ppm. HRMS $\{C_2H_5CB_{11}H_{11}^-\}$ = calculated: 171.23482 m/z, found: 171.23715 m/z.

Mg**2c**: $^{11}B\{^{1}H\}$ NMR (128 MHz, D_6-Acetone): ∂ = -7.32 ppm (bs, 1H, B-H), -10.89 ppm (bs, 10H, B-H). ^{11}B NMR (128 MHz, D_6-Acetone): ∂ = -6.80, -7.86, -10.33, -11.44 ppm. ^{1}H NMR (400 MHz, D_6-Acetone): ∂ = 3.47 (s, 12H), 3.29 (s, 18H), 1.76 (t, 4H), 1.32 (m, 4H) 0.75 (t, 6H) ppm. ^{13}C NMR (100 MHz, D_6-acetone): ∂ = 72.35, 70.81, 58.78, 42.74, 24.09, 14.41 ppm. HRMS $\{C_3H_7CB_{11}H_{11}^-\}$ = calculated: 185.25047 m/z, found: 185.25239 m/z.

Mg**2d**: $^{11}B\{^1H\}$ NMR (128 MHz, D_6-Acetone): ∂ = -10.09 ppm (bs, 1H, B-H), -13.61 ppm (bs, 10H, B-H). ^{11}B NMR (128 MHz, D_6-Acetone): ∂ = -8.45, -9.15, -11.95, -12.65 ppm. ^1H NMR (400 MHz, D_6-Acetone): ∂ = 3.47 (s, 12H), 3.29 (s, 18H), 1.79 (t, 4H), 1.29 (m, 4H) 1.16 (m, 4H), 0.83 (t, 6H) ppm. ^{13}C NMR (100 MHz, D_6-acetone): ∂ = 71.56, 69.99, 57.91, 39.32, 32.39, 22.56, 13.36 ppm. HRMS $\{C_4H_9CB_{11}H_{11}^-\}$ = calculated: 199.26612 m/z, found: 199.2681 m/z.

Mg**2e**: $^{11}B\{^1H\}$ NMR (128 MHz, D_6-Acetone): ∂ = -7.21 ppm (bs, 1H, B-H), -10.74 ppm (bs, 10H, B-H). ^{11}B NMR (128 MHz, D_6-Acetone): ∂ = -6.69, -7.74, -10.18, -11.29 ppm. ^1H NMR (400 MHz, D_6-Acetone): ∂ = 3.47 (s, 12H), 3.29 (s, 18H), 1.79 (t, 4H), 1.24 (m, 8H) 1.14 (m, 4H), 0.85 (t, 6H) ppm. ^{13}C NMR (100 MHz, D_6-acetone): ∂ = 71.99, 70.46, 58.35, 39.96, 32.20, 30.27, 22.73, 13.87 ppm. HRMS $\{C_5H_{11}CB_{11}H_{11}^-\}$ = calculated: 213.28177 m/z, found: 213.2833 m/z.

Mg**2f**: $^{11}B\{^1H\}$ NMR (128 MHz, D_6-Acetone): ∂ = -7.06 ppm (bs, 1H, B-H), -10.75 ppm (bs, 10H, B-H). ^{11}B NMR (128 MHz, D_6-Acetone): ∂ = -6.55, -7.60, -10.19, -11.30 ppm. ^1H NMR (400 MHz, D_6-Acetone): ∂ = 3.47 (s, 12H), 3.29 (s, 18H), 1.72 (d, 4H), 1.65 (m, 2H) 0.84 (d, 12H), 0.85 (t, 6H) ppm. ^{13}C NMR (100 MHz, D_6-acetone): ∂ = 71.37, 69.45, 57.81, 48.44, 28.32, 23.30 ppm. HRMS $\{C_4H_9CB_{11}H_{11}^-\}$ = calculated: 199.26612 m/z, found: 199.26872 m/z.

Mg**2g**: $^{11}B\{^1H\}$ NMR (128 MHz, D_6-Acetone): ∂ = -8.86 ppm (bs, 1H, B-H), -12.41 ppm (bs, 10H, B-H). ^{11}B NMR (128 MHz, D_6-Acetone): ∂ = -9.02, -10.08, -12.57, -13.68 ppm. ^1H NMR (400 MHz, D_6-Acetone): ∂ = 3.48 (s, 12H), 3.29 (s, 18H), 1.80 (t, 4H), 1.35 (m, 2H) 1.21 (m, 4H), 0.80 (d, 12H) ppm. ^{13}C NMR (100 MHz, D_6-acetone): ∂ = 71.37, 69.45, 57.81, 48.44, 28.32, 23.30 ppm. HRMS $\{C_5H_{11}CB_{11}H_{11}^-\}$ = calculated: 213.28177 m/z, found: 213.2842 m/z.

Electrolyte Formulation
0.8 M solutions of Mg**2g**/DME were obtained by dissolution of $([R\text{-}CB_{11}H_{11}]^-)_2[Mg(DME)_3]^{2+}$ (800 mg, 1.1 mmol) in dimethoxyethane (0.72 mL). The solution was allowed to stir for 1 hour or until complete dissolution of the salt was achieved. The resulting electrolyte was filtered through a glass microfiber filter (Whatman, F Grade) and stored in a 10 mL Teflon sealed Schlenk flask and used without further purification. H_2O content of the electrolytes used in this study were verified to be <10 ppm by Karl Fisher titration prior to use.

Electrode Fabrication
Chevrel phase $Cu_2Mo_6S_8$ was first synthesized via solid state reaction by heating stoichiometric mixture of elemental Cu, Mo and S powders. Stoichiometric amounts of Cu powder (Alfa Aesar, 10 micron, 99.9%), Mo powder (Alfa Aesar, 2-4 micron, 99.9%) and S powder (Sigma Aldrich, 99.5-100%) were thoroughly mixed and sealed in an evacuated quartz tube. The quartz tube was subsequently heated in a muffle furnace. The temperature ramp program was set as follows: The temperature was ramped up to 450 °C (1 °C min^{-1}) and held for 24 h. The temperature was then ramped up to 700 °C (1 °C min^{-1}) and held for another 24 h. The temperature was thereafter ramped up again to 1050 °C (1 °C min^{-1}) and held for 48 h. Finally, the furnace was allowed to cool down to room temperature naturally. The synthesized Chevrel phase $Cu_2Mo_6S_8$ then underwent a chemical leaching process to yield the final product of Mo_6S_8 as follows: $Cu_2Mo_6S_8$ was added into 20 ml 6M HCl solution. Oxygen was bubbled into the solution for 8 hours while stirring. Following the reaction, the obtained Mo_6S_8 was centrifuged, washed with adequate amount of deionized water, and dried in vacuum oven at 50 °C overnight. The resulting product was characterized by SEM/EDX and powder XRD to verify purity. The electrode slurry was made by mixing 80 wt.% Mo_6S_8, 10 wt.% carbon black, and 10 wt.% PVDF in N-Methyl-2-pyrrolidone (NMP) solution via a mechanical mixer for 15 min. The slurry was coated on thin Al foil with Mo_6S_8 loading of 1.5 mg cm^{-2}.

Raman Spectroscopy
Raman spectra were acquired on a confocal Raman microspectrometer (LabRam Evolution, Horiba, Japan) following excitation at 532 nm. An 1800 groove/mm grating was selected to provide < 1cm^{-1} spectral resolution. A 50x long-working distance objective (LMPlanFL, Olympus, Japan, wd: 10.6 mm, na: 0.5) was used to image the sample (spot size: $\leq$1.3 mm). Samples were prepared in a glovebox under nitrogen atmosphere and sealed in borosilicate glass NMR tubes.

Electrochemistry and Device Fabrication
Three-electrode cyclic voltammetry was carried out in a 200 mL glass Gamry 5 neck cell with a platinum (Gamry, 99.999%) working electrode (3 mm disc) and two Mg plates (0.25 mm thickness, 99.9%, Alfa Aesar) as the reference electrode and counter electrode, respectively. The Pt working electrode was polished with alumina particle (0.05 µm) water dispersion on a polishing pad, sonicated in ethanol, and dried at least 12 hours under vacuum prior to each experiment. Overpotential for 3-electrode experiments was determined by the potential difference between onset of reduction and 0 V vs. $Mg^{2+/0}$. Two-electrode symmetric (Mg||Mg) and half cell (Mg||Cu and Mg||Mo_6S_8) experiments in both 2016 and 2032 stainless steel casing were employed for coin cell fabrication and glass microfiber was used as a separator (Whatman, F grade). Voltage hysteresis

for constant current experiments undertaken with Mg||Cu was determined by the potential difference between the reduction/oxidation plateau. Magnesium metal (Sigma Aldrich, 99.9 %) was obtained as a foil, polished with 2000 grain sandpaper (3M), and punched into disks 15.75 mm in diameter. Mg anodes submerged in hexane were sonicated for 1 hour, washed with 5 mL of hexane (3x), and dried *in-vacuo* for 2 hours prior to use. Identical Mg metal electrodes were used as the working and counter electrode. Chevrel Phase molybdenum sulfide (Mo_6S_8) wasprepared following previously reported procedures and characterized via SEM/EDX and powder XRD following synthesis.[3] Cells were assembled in an argon-filled glove box (>0.5 ppm H2O, >1 ppm O2). Galvanostatic charge-discharge cycling tests were performed by a battery cycler system (MACCOR). Electrochemical Impedance Spectroscopy (EIS) of Mg**2g**/DME was conducted in a 2-electrode T-cell apparatus consisting of electrodes of known surface area and separation distance over a 25°C-60°C temperature range. Copper rods of known surface area were separated at a fixed path length and used to measure electrolyte resistance. Two copper rods were placed on two sides of the T cell and wrapped with Teflon tape to prevent leaking. The cell was filled with electrolyte Mg**2g**/DME until the electrodes were fully submerged. Finally, the cell was sealed at the top with a third copper rod. The resistance of the electrolyte was measured by EIS with frequency from 30 kHz to 10 Hz under voltage of 10 mV rms. The resistance was determined as the first data point intercepted with the x axis (Figure S37). Ionic conductivity was calculated from ohm's law where σ is conductivity, ρ is resistivity, L is the length between the electrodes across the electrolyte, A is the surface area of electrodes, and R is the resistance of the electrolyte:

$$\sigma = \frac{1}{\rho} = \frac{L}{A.R}$$

Surface Characterization

Samples were prepared via electrodeposition of magnesium metal in a Mg||Cu 2032 coin cell configuration followed by disassembly in an argon-filled glovebox. Copper electrodes were washed liberally with anhydrous DME and allowed to dry under vacuum for 12 hours. The surface morphology and elemental composition of the samples were characterized with scanning electron microscopy (SEM, Nova Nano S450) and energy-dispersive X-ray (EDX) spectroscopy.

Crystal Structure Determination

Single crystals suitable for X-ray diffraction were grown by slow diffusion of hexane into dimethoxyethane. A colourless crystal (plate, approximate dimensions 0.49 × 0.16 × 0.12 mm3) was placed onto the tip of a MiTeGen pin and mounted on a Bruker Venture D8 diffractometer equipped with a PhotonIII detector at 180.00 K. The data collection was carried out using Mo Kα radiation (λ = 0.71073 Å, ImS micro-source) with a frame time of 15 seconds and a detector distance of 60 mm. A collection strategy was calculated and complete data to a resolution of 0.84 Å with a redundancy of 5.3 were collected. The frames were integrated with the Bruker SAINT[4] software package using a narrow-frame algorithm to a resolution of 0.84 Å. Data were corrected for absorption effects using the Multi-Scan method (SADABS).[5] Please refer to Table S1 for additional crystal and refinement information.

The space group $Pna2_1$ was determined based on intensity statistics and systematic absences. The structure was solved using the SHELX suite of programs[6, 7] and refined using full-matrix least-squares on F^2 within the OLEX2 suite.[8] And intrinsic phasing solution was calculated, which provided most non-hydrogen atoms from the E-map. Full-matrix least squares / difference Fourier cycles were performed, which located the remaining non-hydrogen atoms. All non-hydrogen atoms were refined with anisotropic displacement parameters. The hydrogen atoms were placed in ideal positions and refined as riding atoms with relative isotropic displacement parameters. The final full matrix least squares refinement converged to R1 = 0.0909 and wR2 = 0.2624 (F^2, all data). The goodness-of-fit was 1.028. On the basis of the final model, the calculated density was 0.944 g/cm^3 and F(000), 3350 e-. The crystals suffer from solvent loss which leads to large anisotropic displacement parameters and solvent accessible voids of 172 Å^3 in the unit cell.

Spectroscopic Characterization

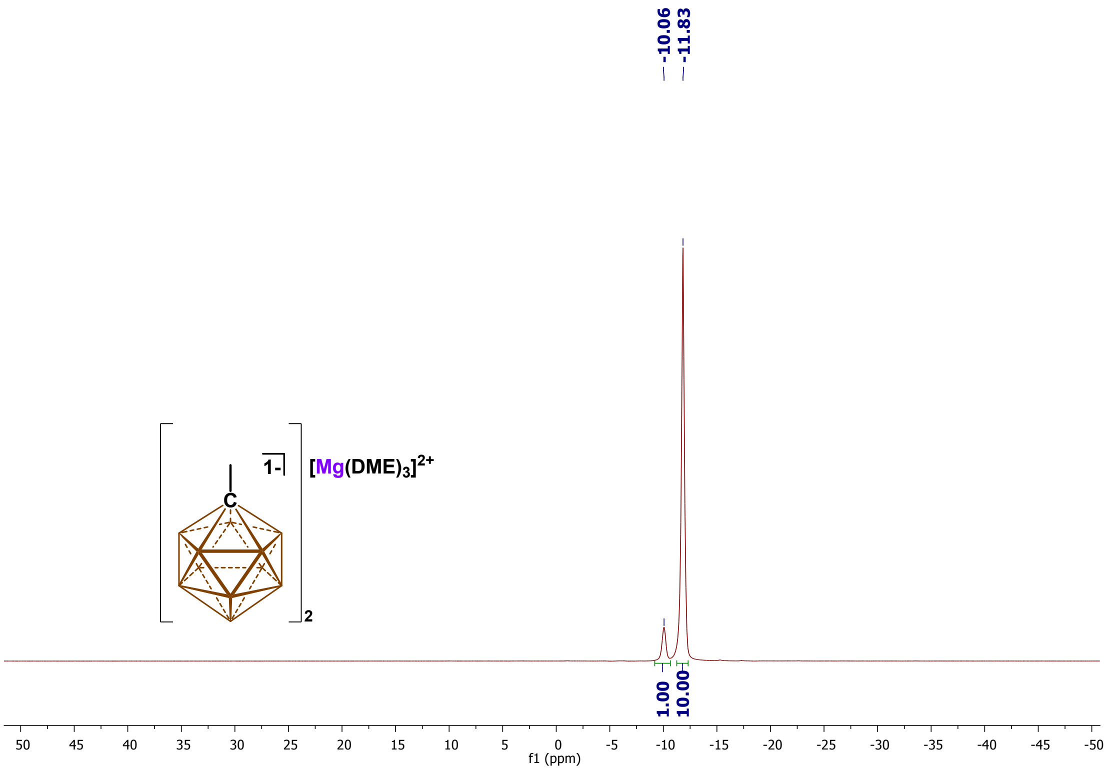

Fig S1. $^{11}B\{^1H\}$ NMR of Mg**2a** in d_6-acetone

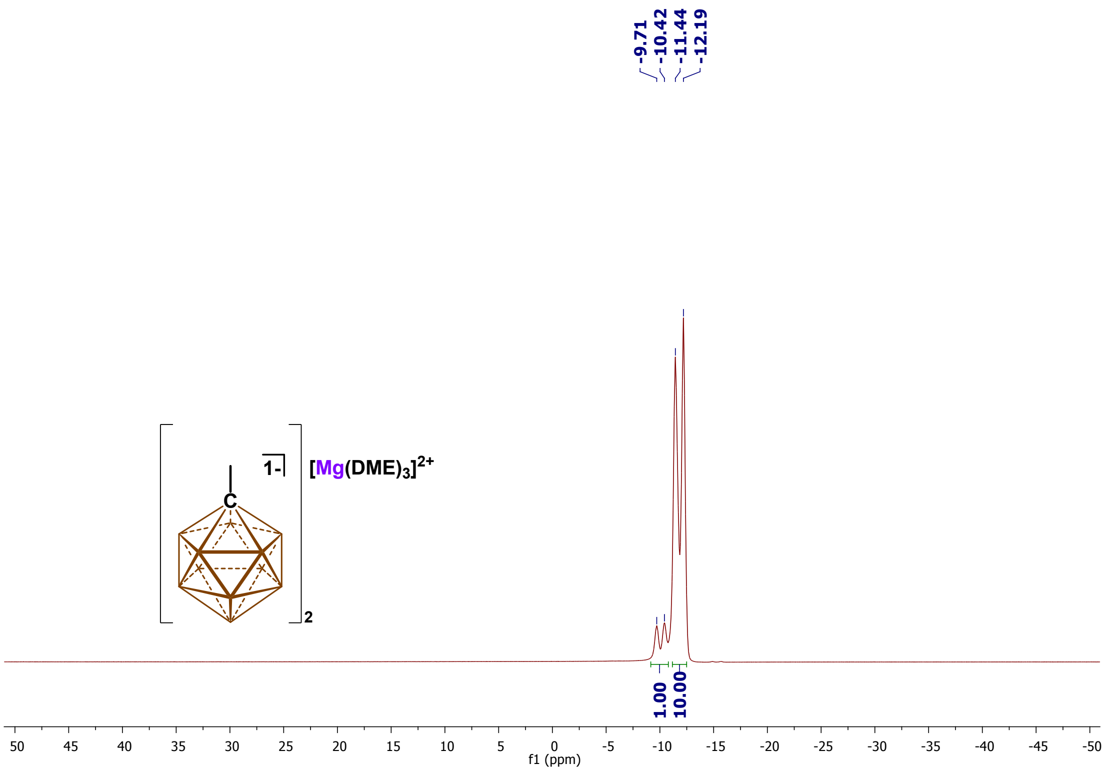

Fig S2. ^{11}B NMR of Mg**2a** in d_6-acetone

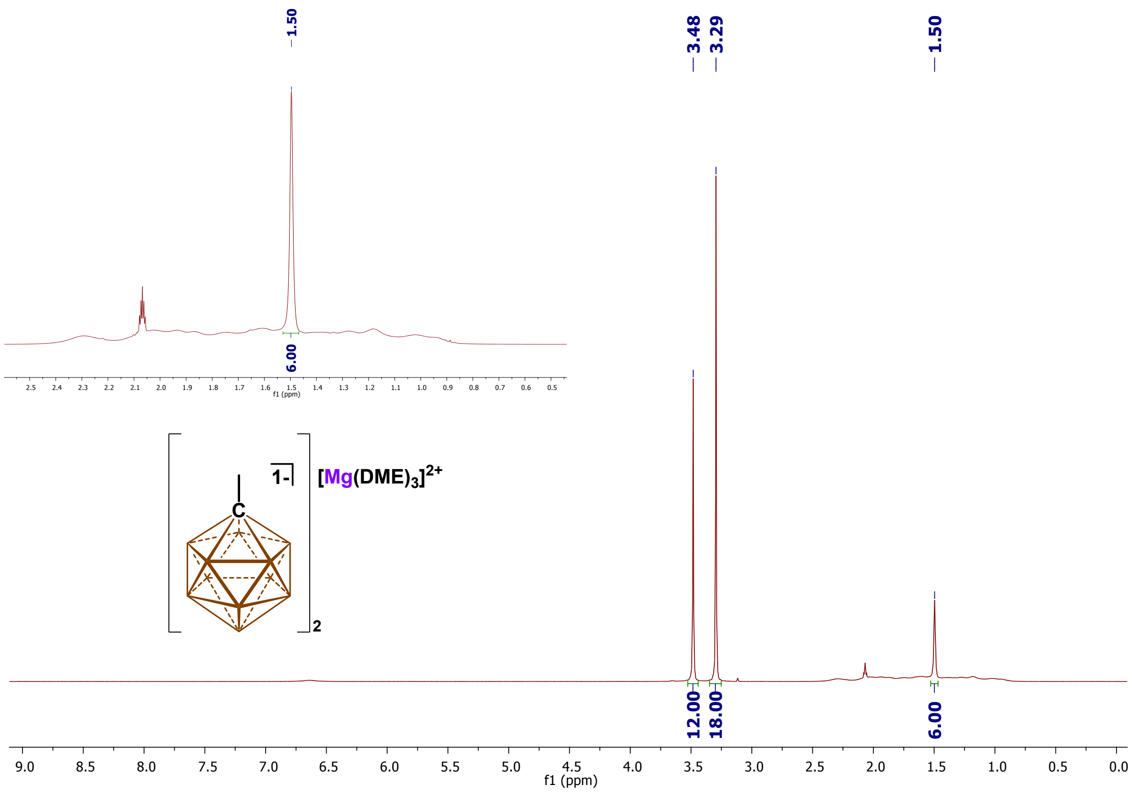

Fig S3. ^{1}H NMR of Mg**2a** in d_6-acetone

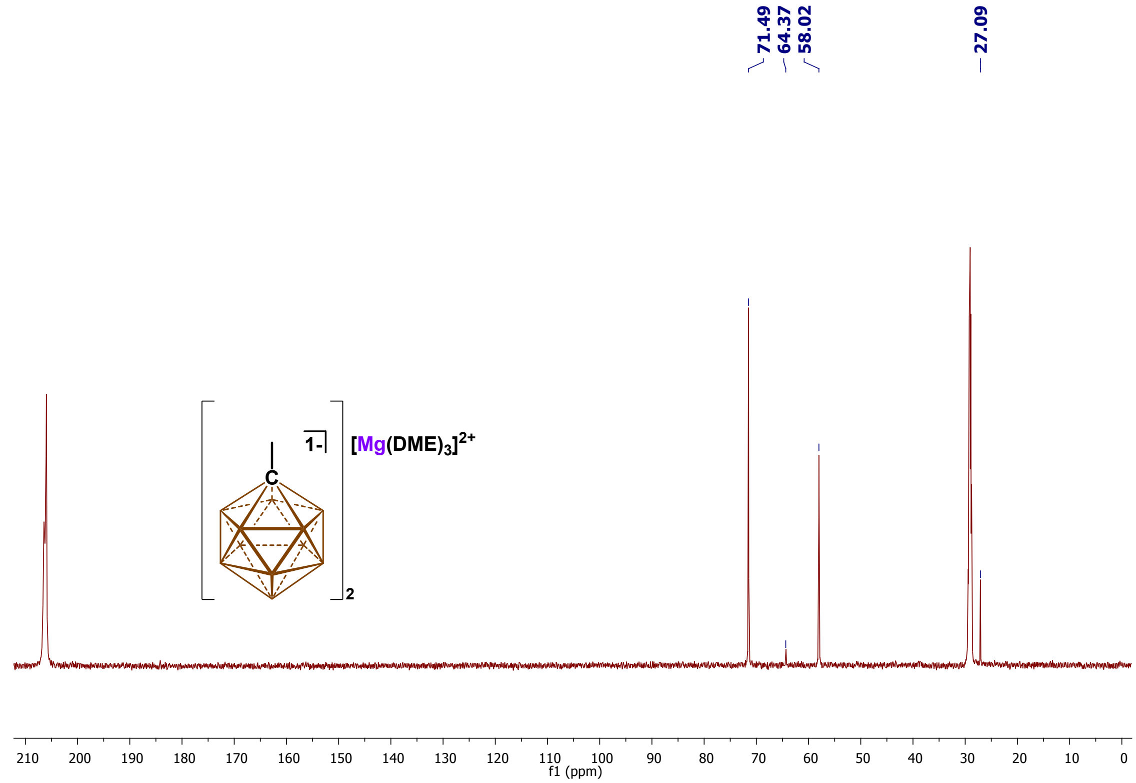

Fig S4. ^{13}C NMR of Mg**2a** in d_6-acetone

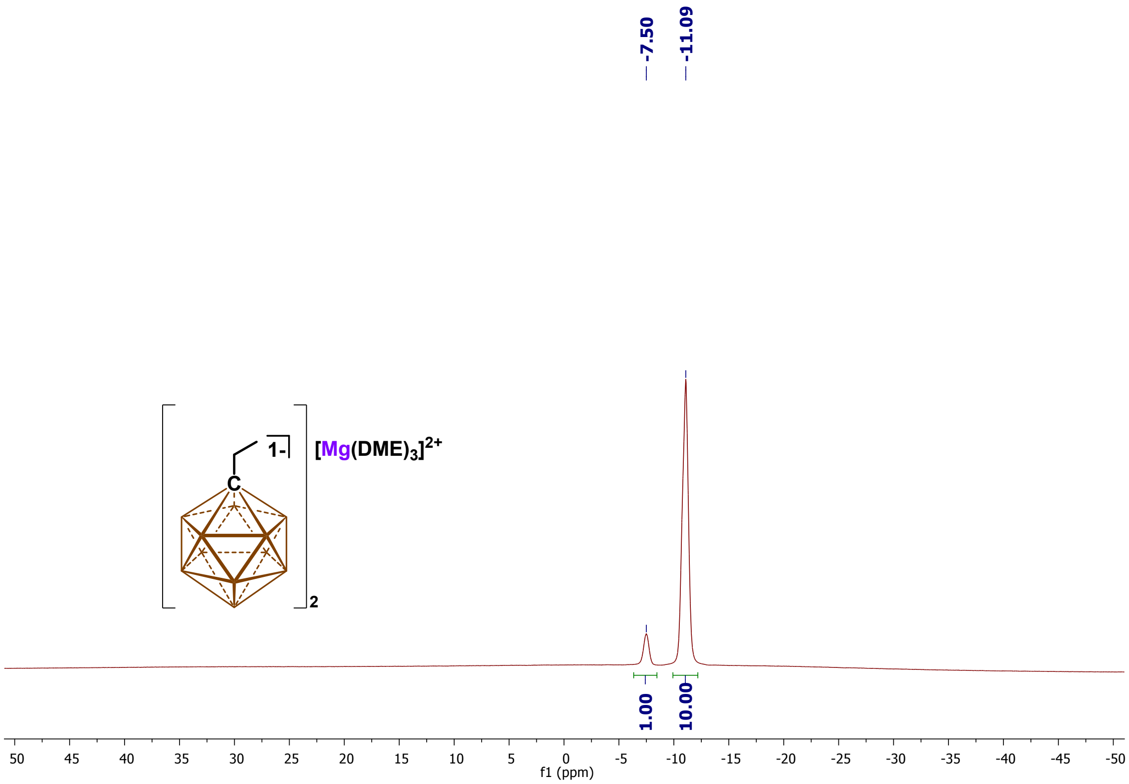

Fig S5. $^{11}B\{^{1}H\}$ NMR of Mg**2b** in d_6-acetone

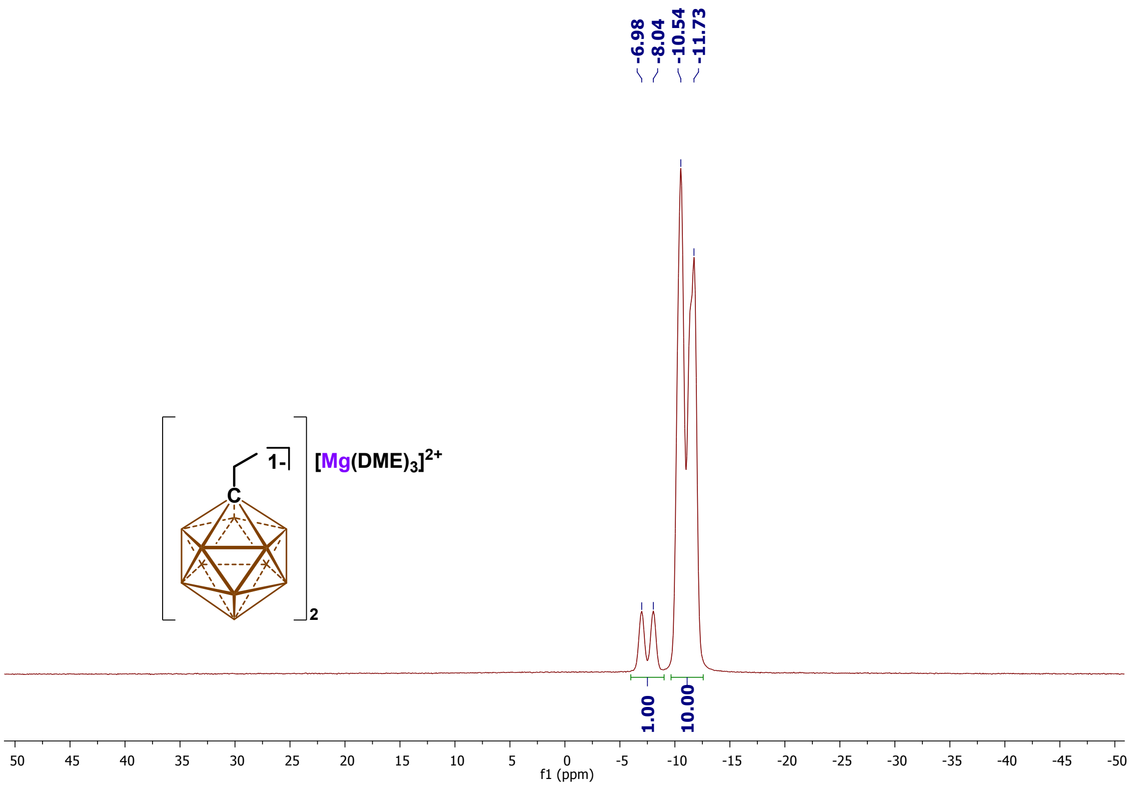

Fig S6. ^{11}B NMR of Mg**2b** in d_6-acetone

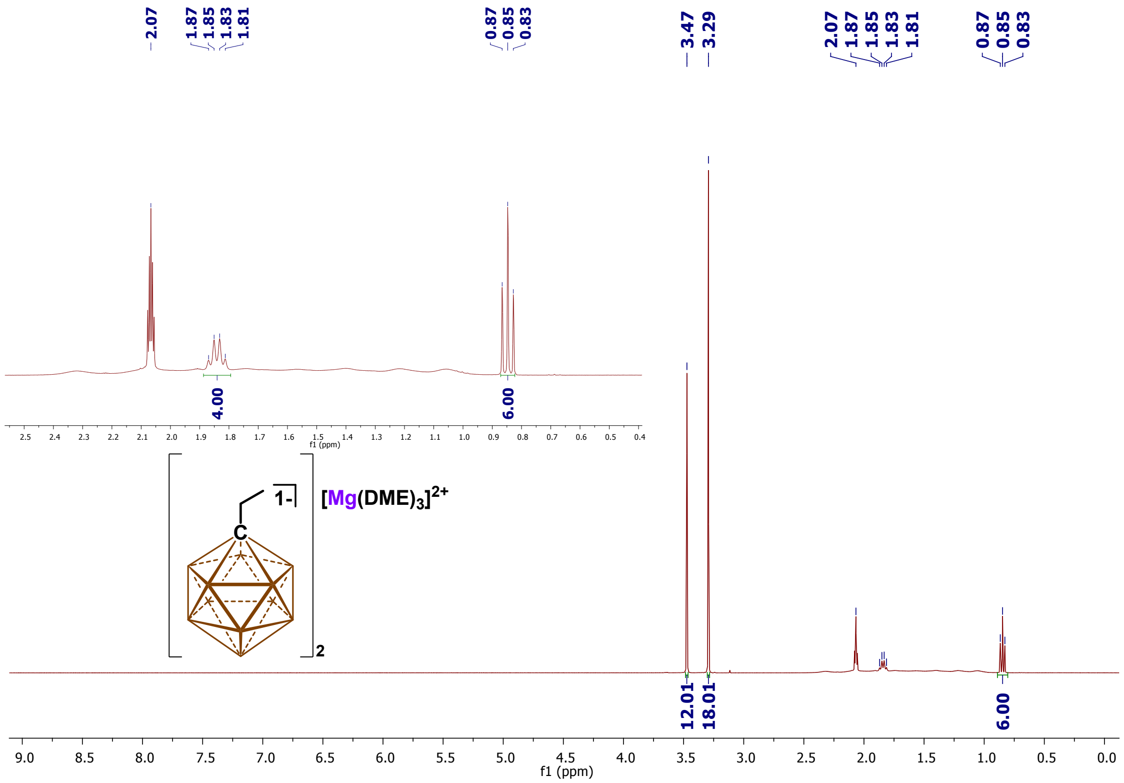

Fig S7. ^{1}H NMR of Mg**2b** in d_6-acetone

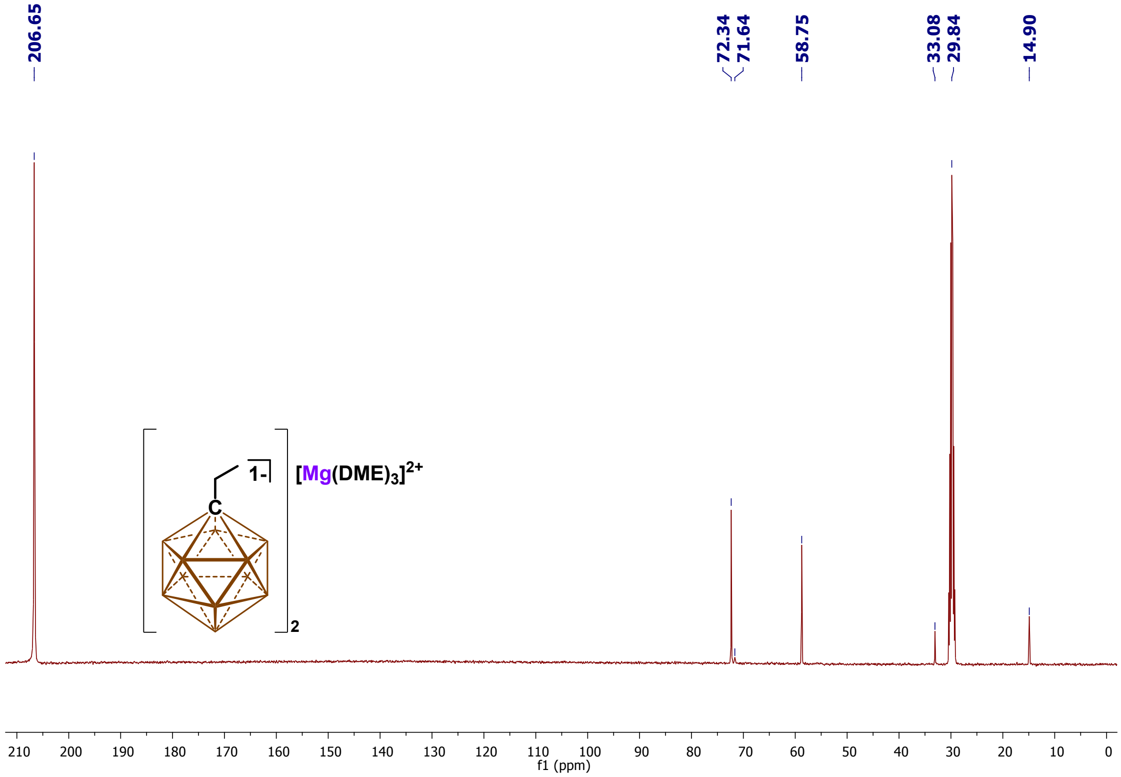

Fig S8. ^{13}C NMR of Mg**2b** in d_6-acetone

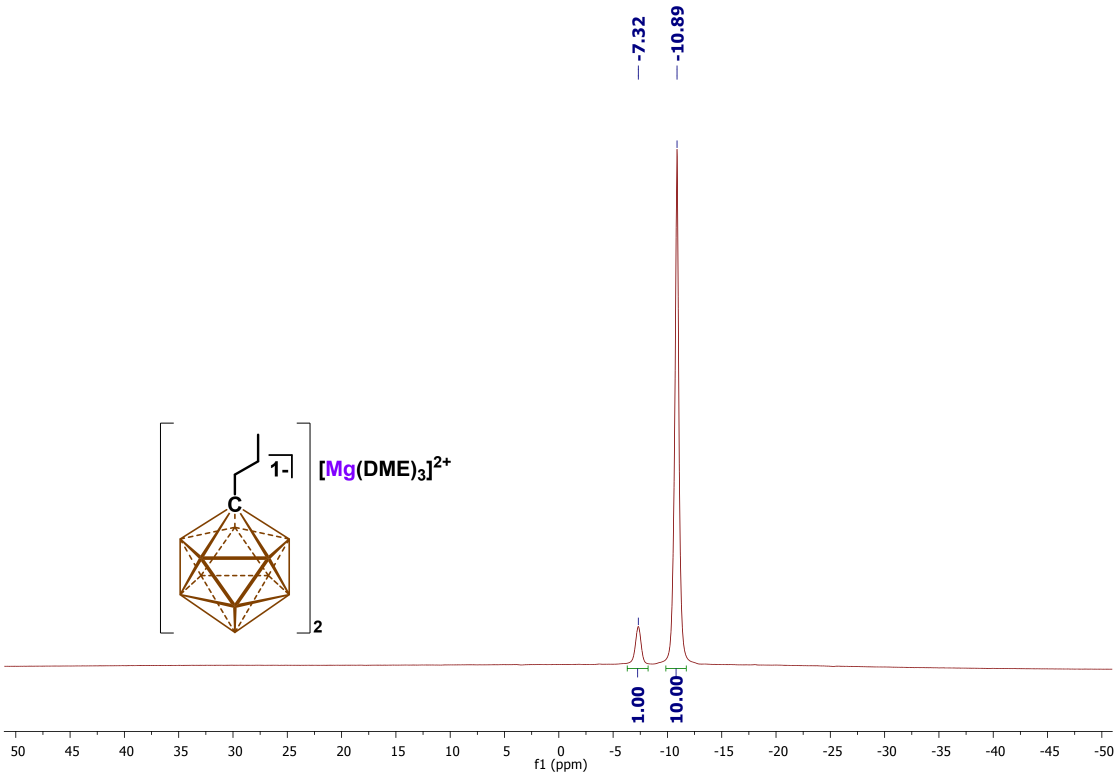

Fig. S9 ^{11}B NMR of Mg**2c** in d_6-acetone

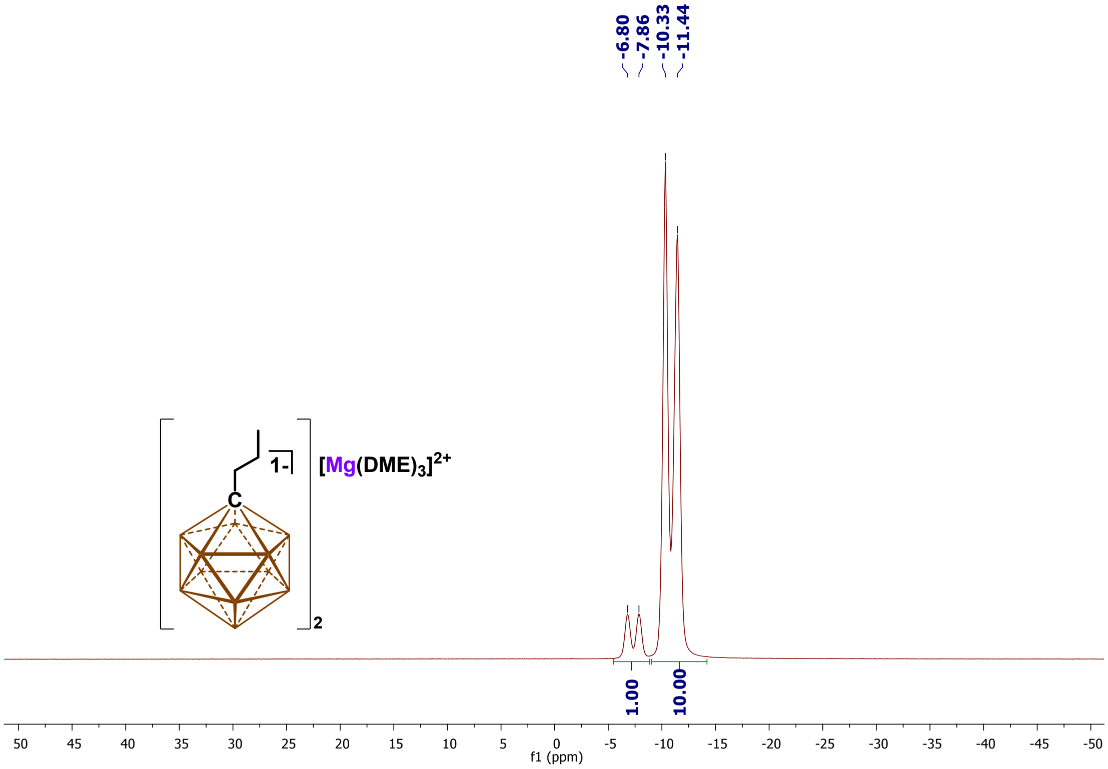

Fig. S10 ^{11}B NMR of Mg**2c** in d_6-acetone

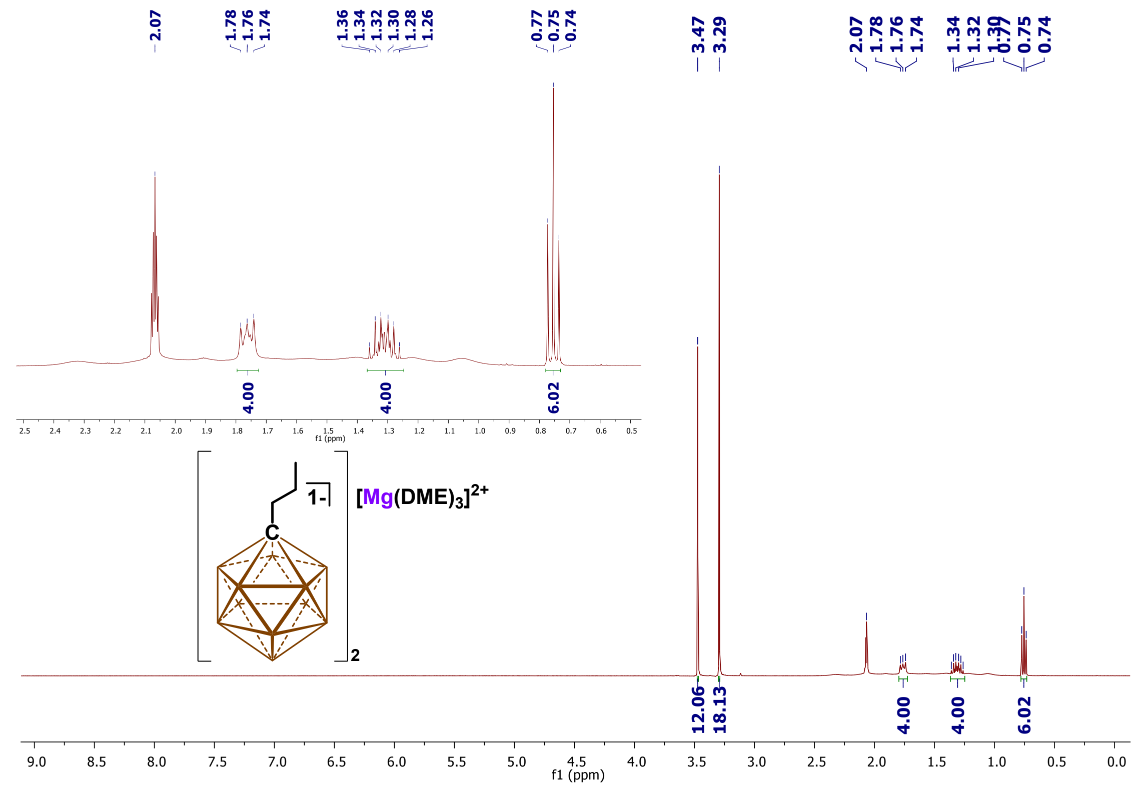

Fig S11. ^{1}H NMR of Mg**2c** in d_6-acetone

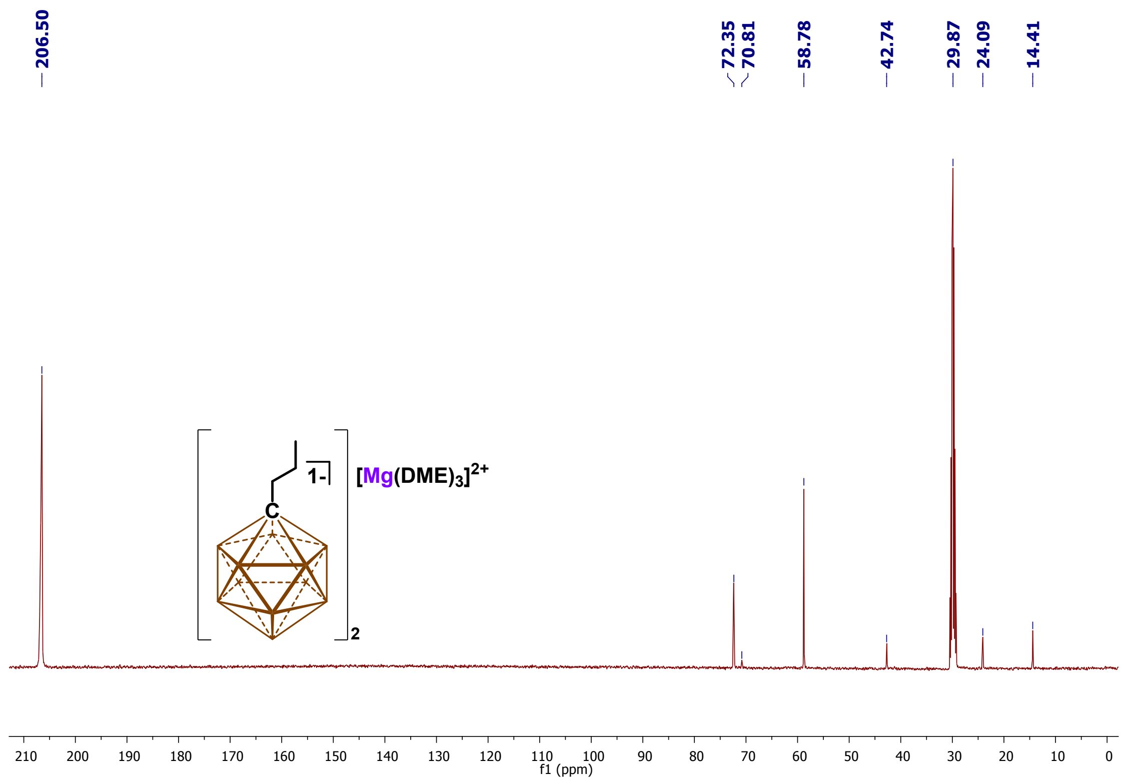

Fig S12. ^{13}C NMR of Mg**2c** in d_6-acetone

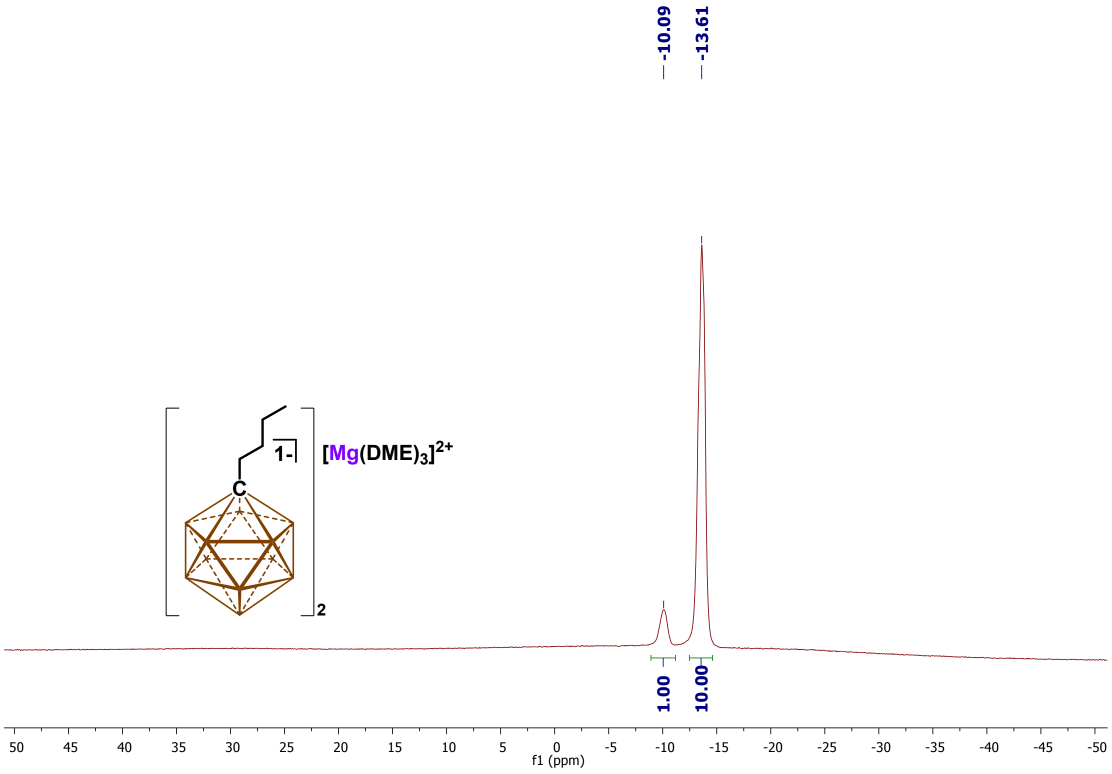

Fig. S13 $^{11}B\{^{1}H\}$ NMR of Mg**2d** in d_6-acetone

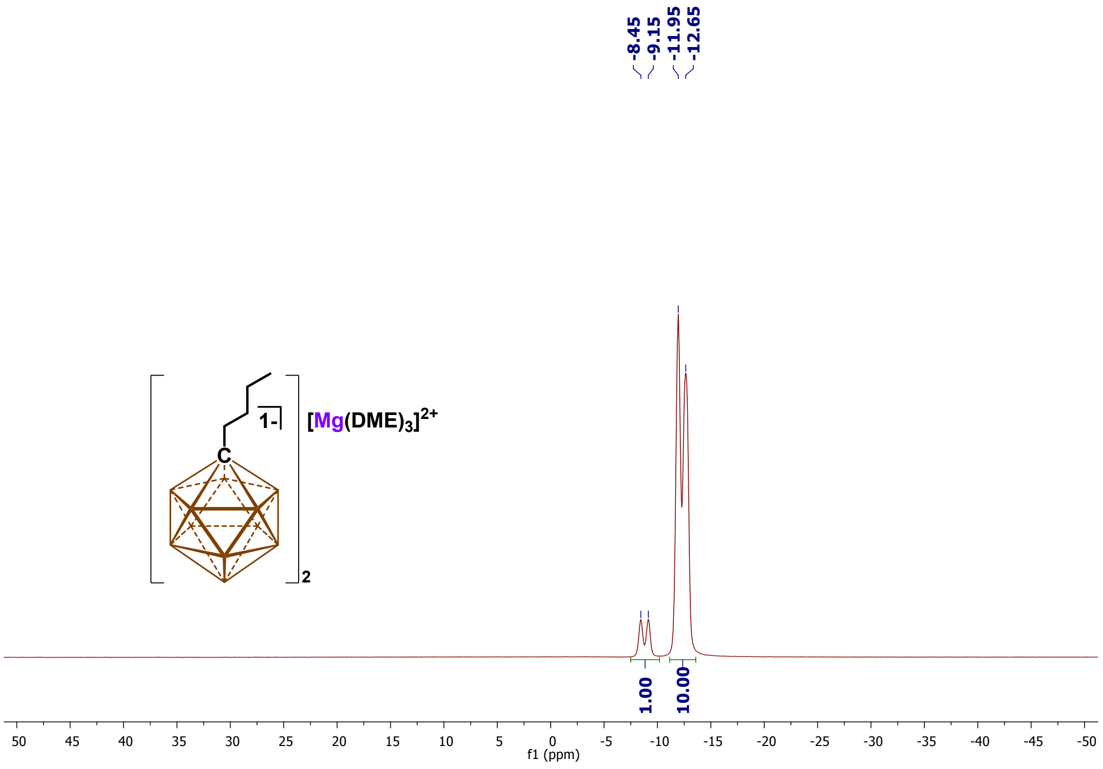

Fig. S14 ^{11}B NMR of Mg**2d** in d_6-acetone

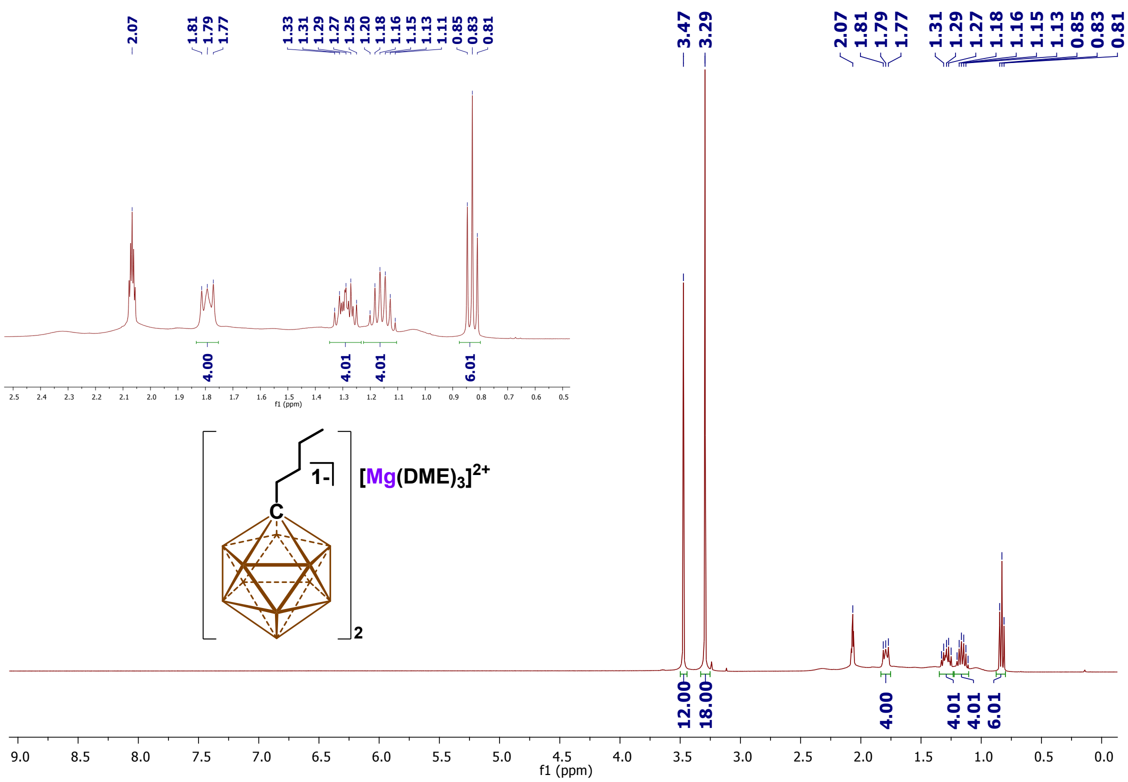

Fig S15. ^{1}H NMR of Mg**2d** in d_6-acetone

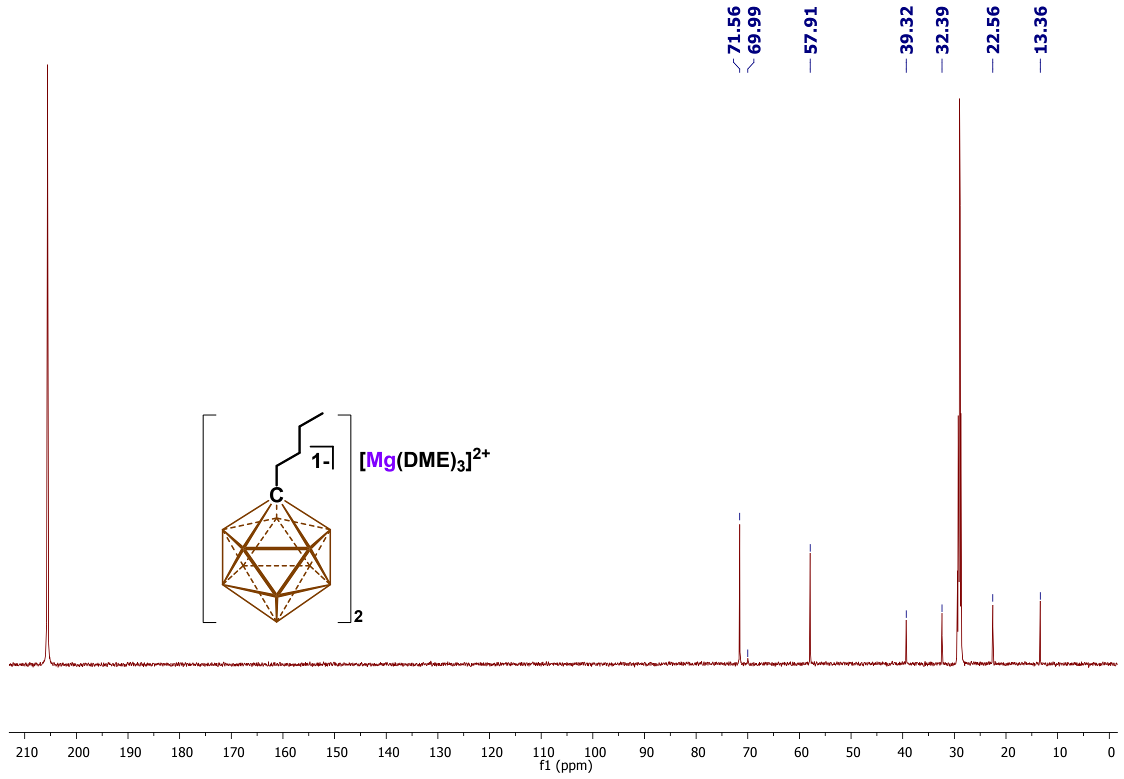

Fig S16. ^{13}C NMR of Mg**2d** in d_6-acetone

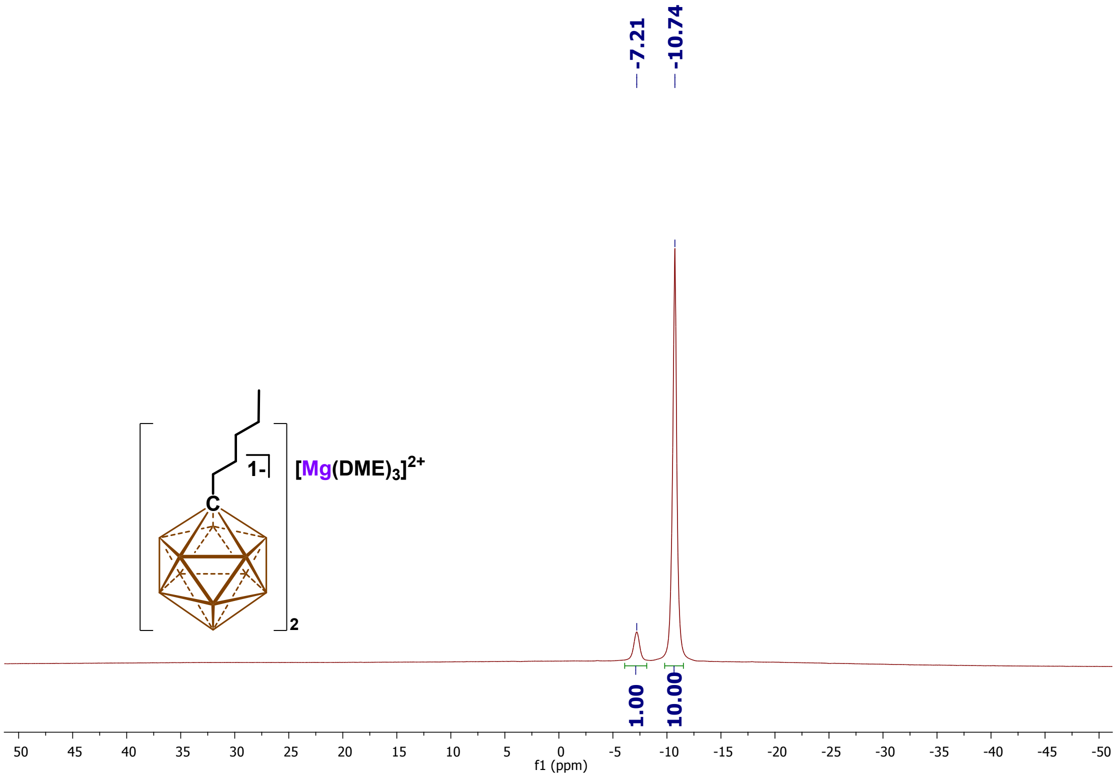

Fig. S17 $^{11}B\{^1H\}$ NMR of Mg**2e** in d_6-acetone

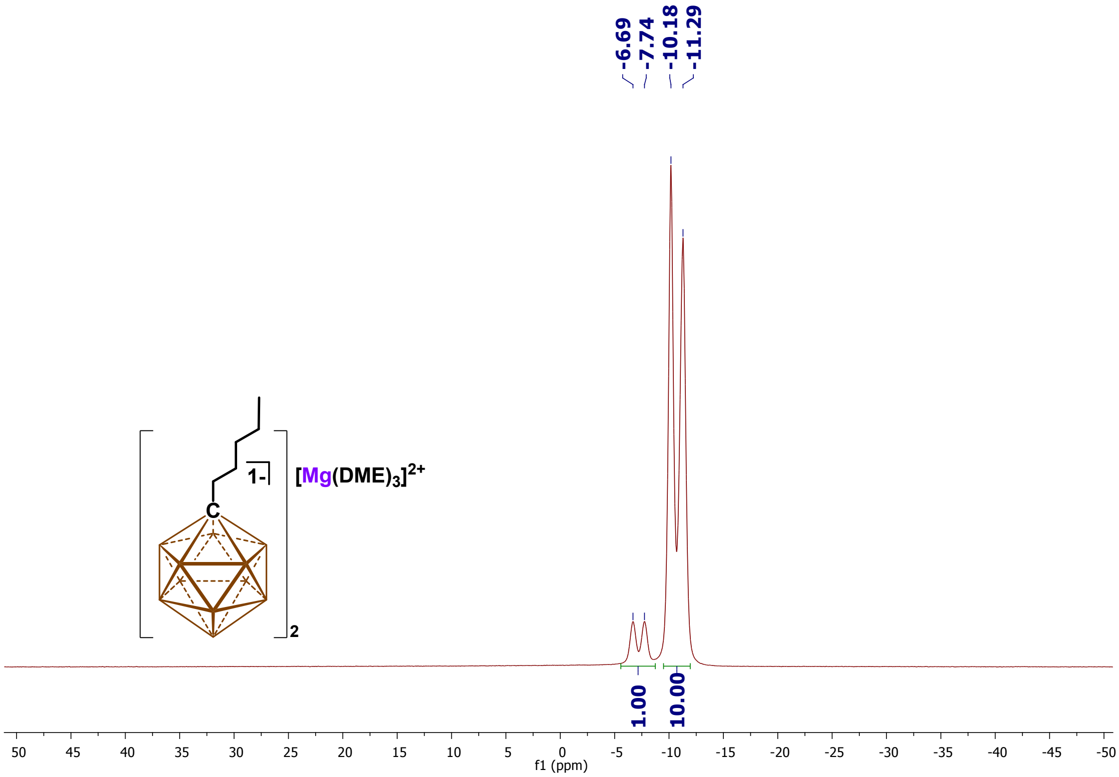

Fig. S18 ^{11}B NMR of Mg**2e** in d_6-acetone

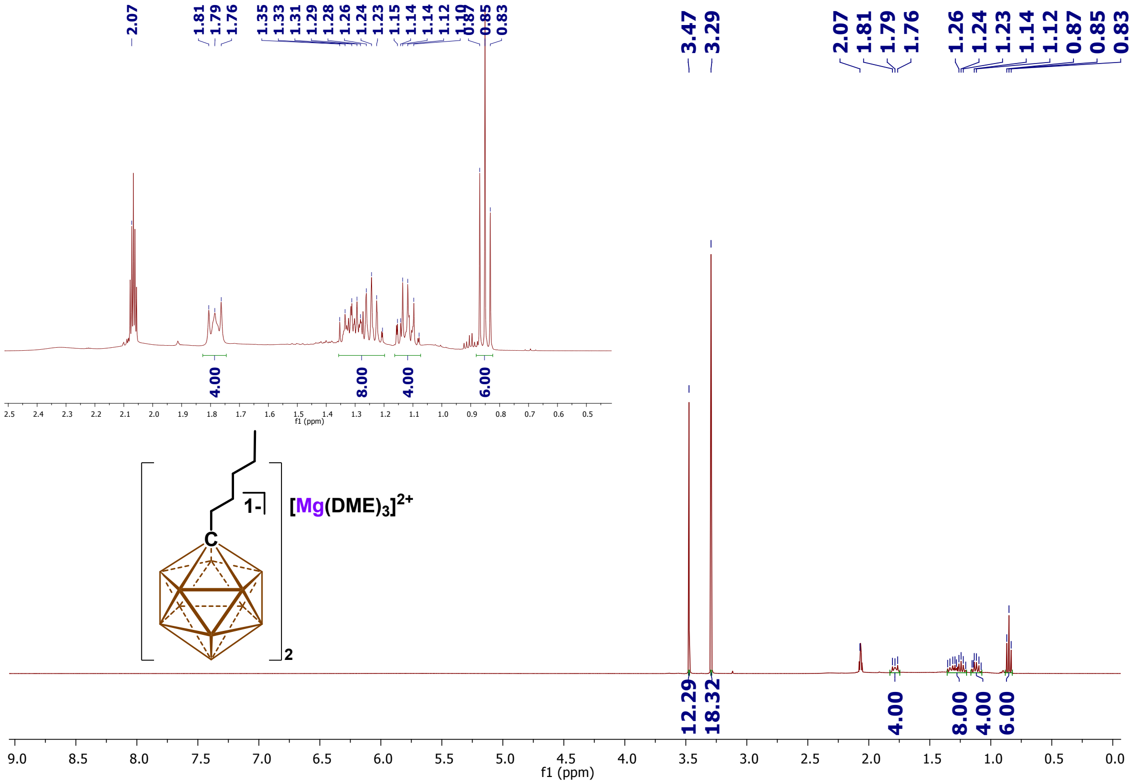

Fig S19. ^{1}H NMR of Mg**2e** in d_6-acetone

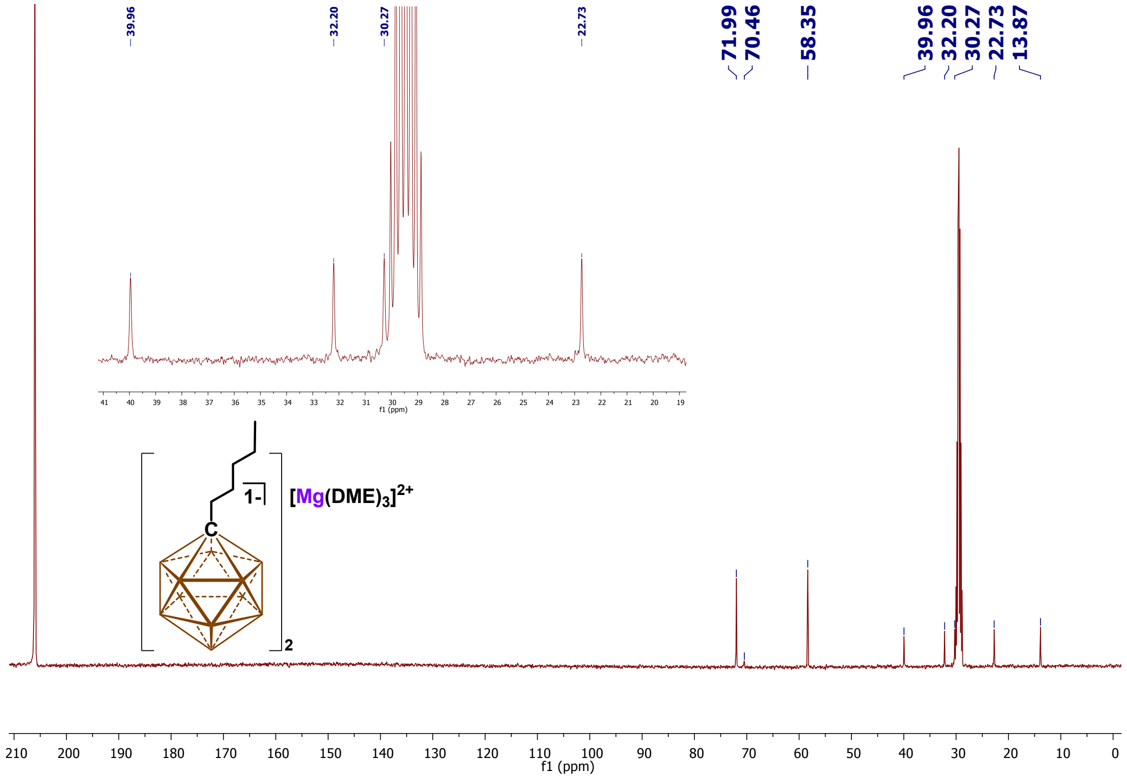

Fig S20. ^{13}C NMR of Mg**2e** in d_6-acetone

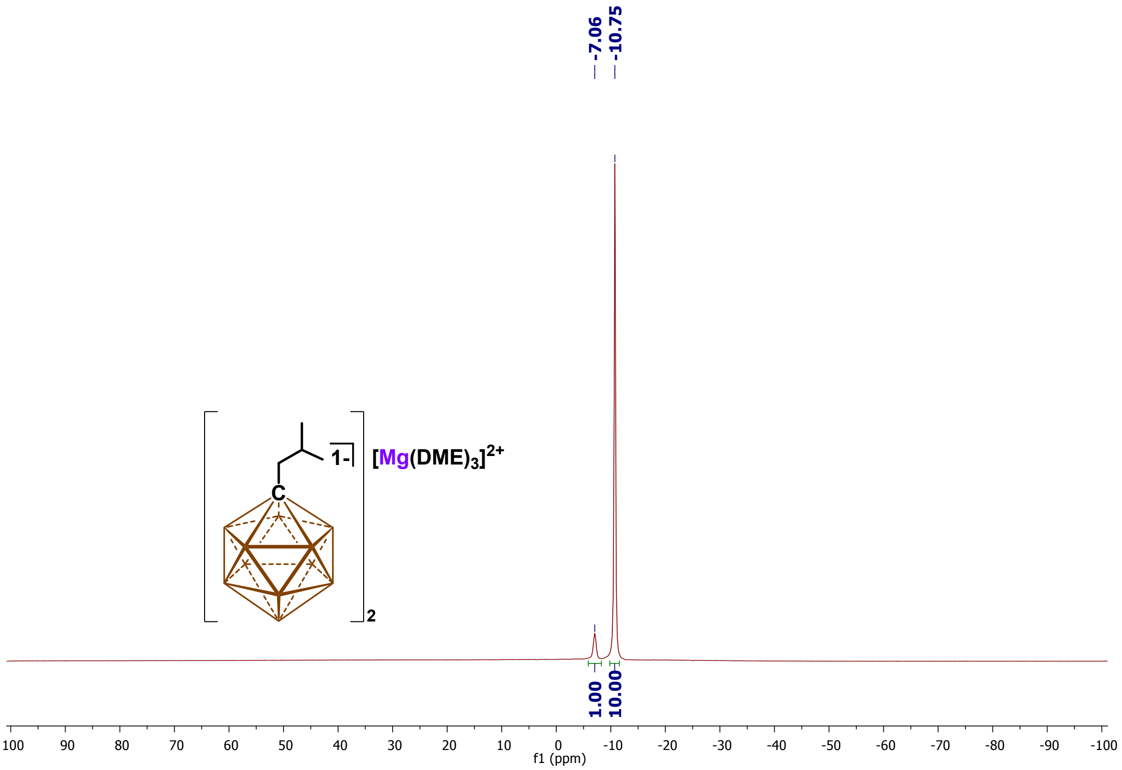

Fig. S21 $^{11}B\{^{1}H\}$ NMR of Mg**2f** in d_6-acetone

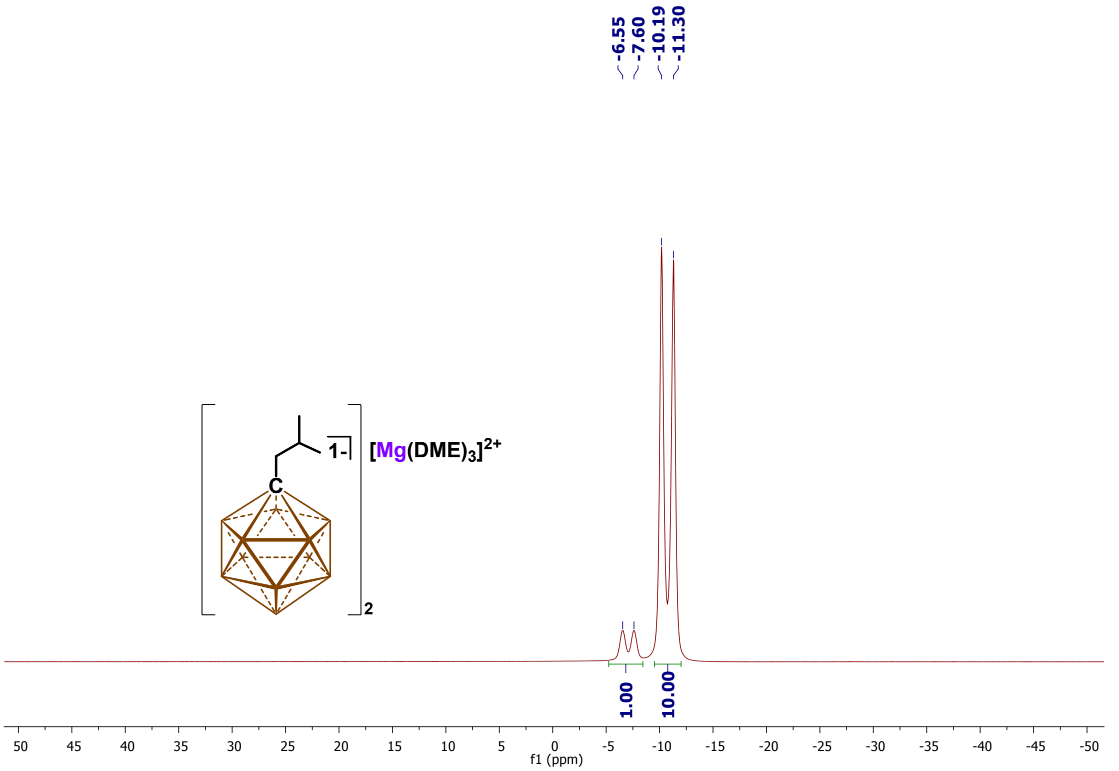

Fig. S22 ^{11}B NMR of Mg**2f** in d_6-acetone

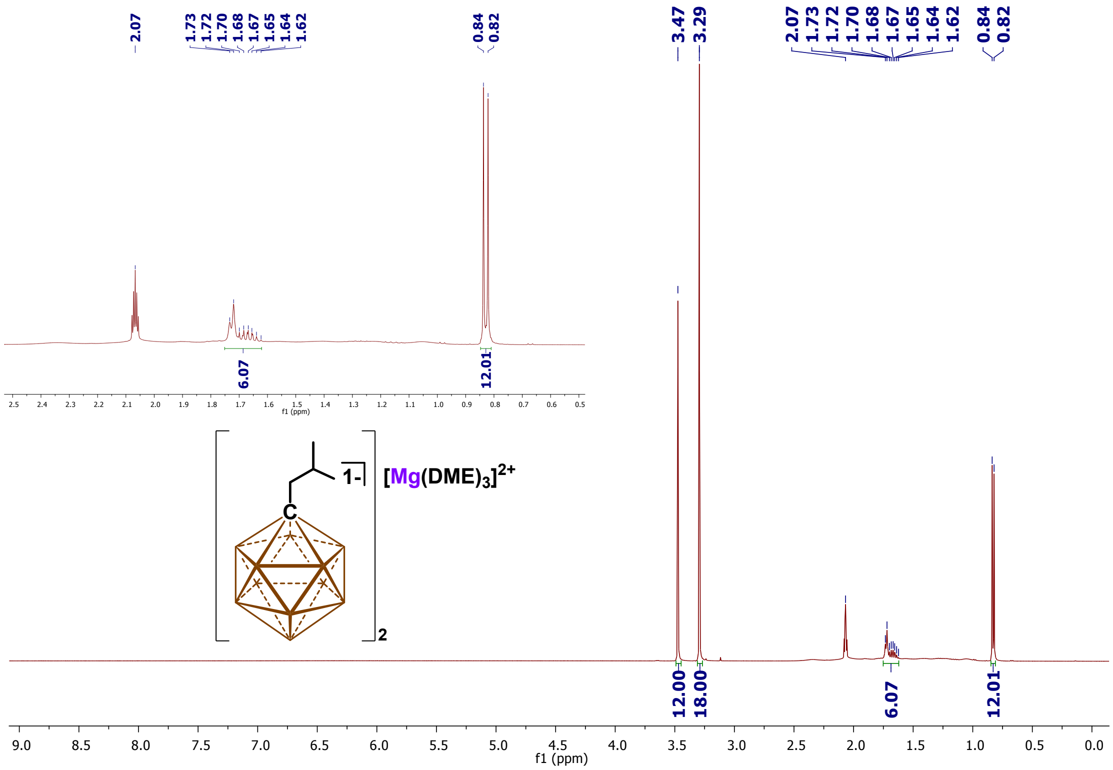

Fig S23. ^{1}H NMR of Mg**2f** in d_6-acetone

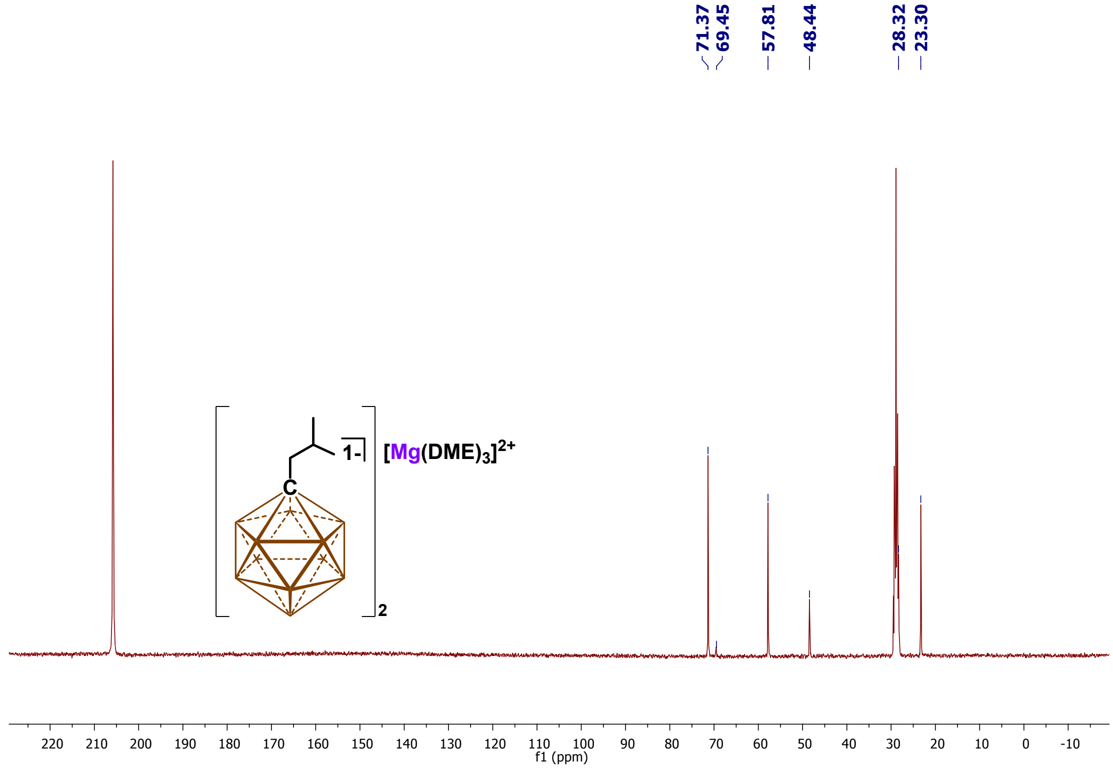

Fig S24. ^{13}C NMR of Mg**2f** in d_6-acetone

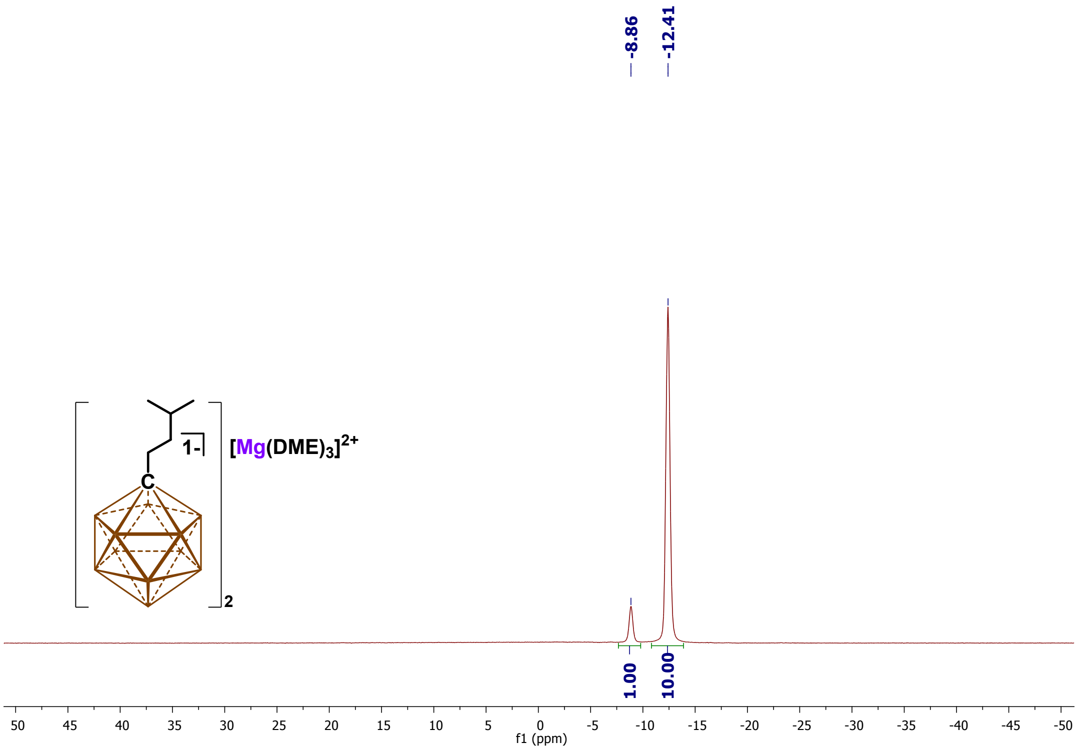

Fig S25. $^{11}B\{^{1}H\}$ NMR of Mg**2g** in d_6-acetone

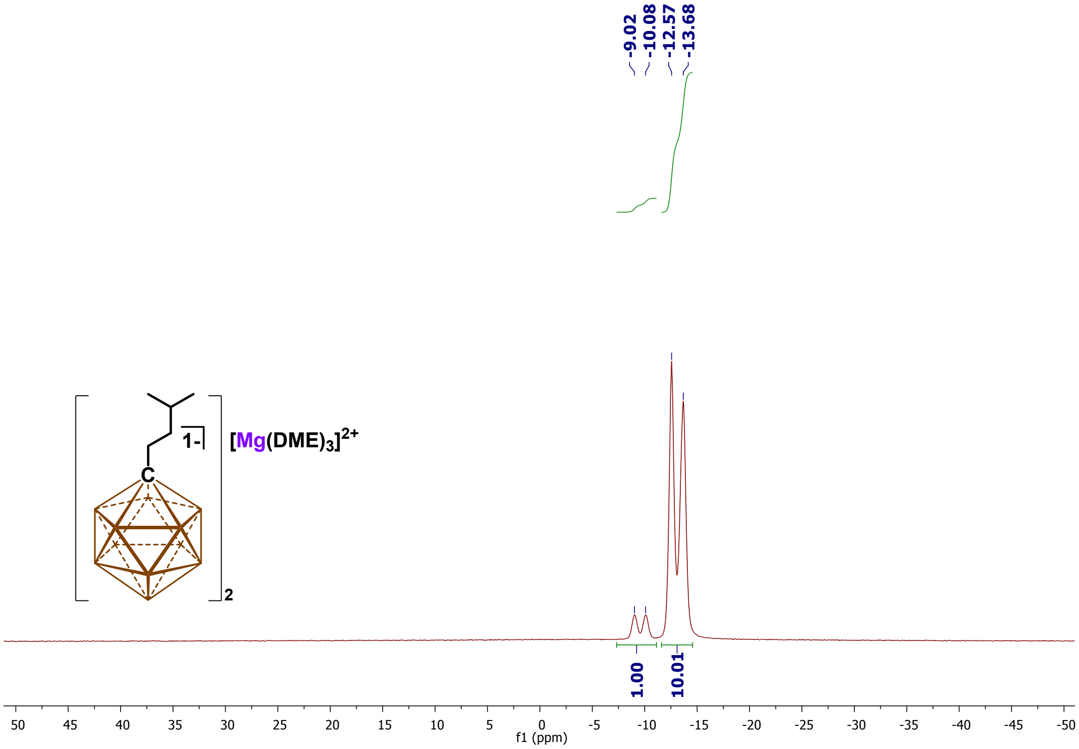

Fig S26. ^{11}B NMR of Mg**2g** in d_6-acetone

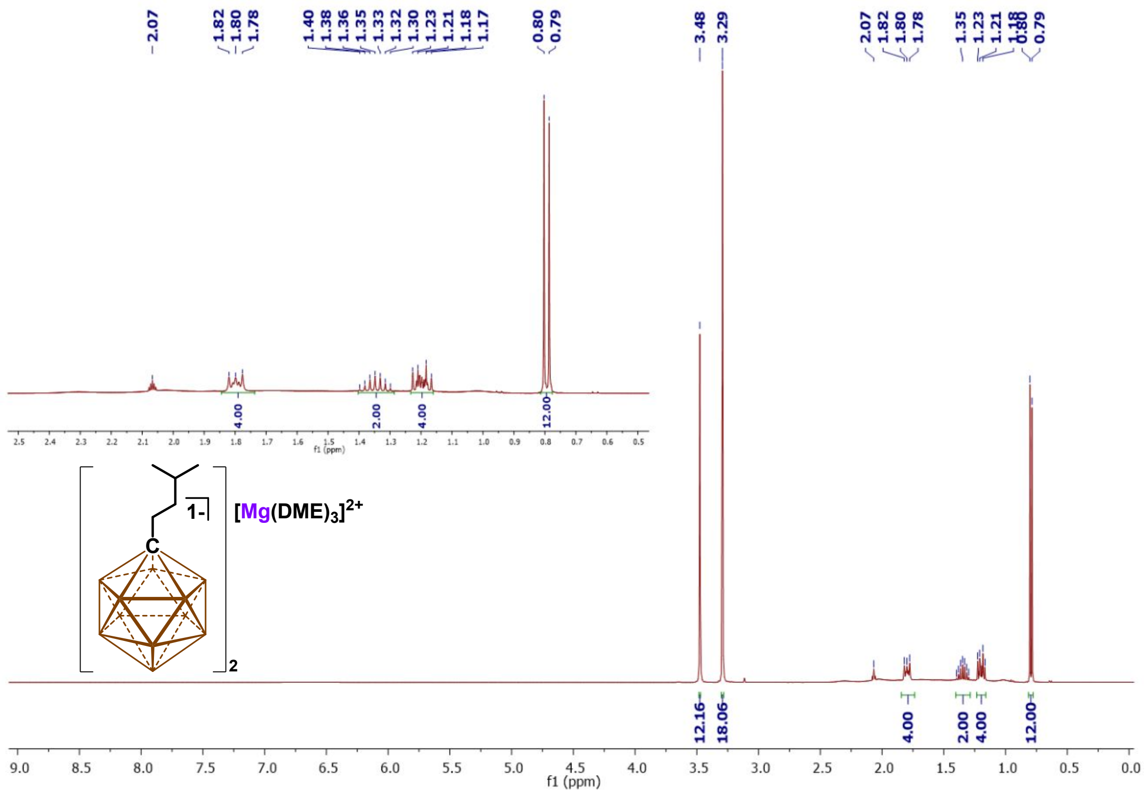

Fig S27. ^{1}H NMR of Mg**2g** in d_6-acetone

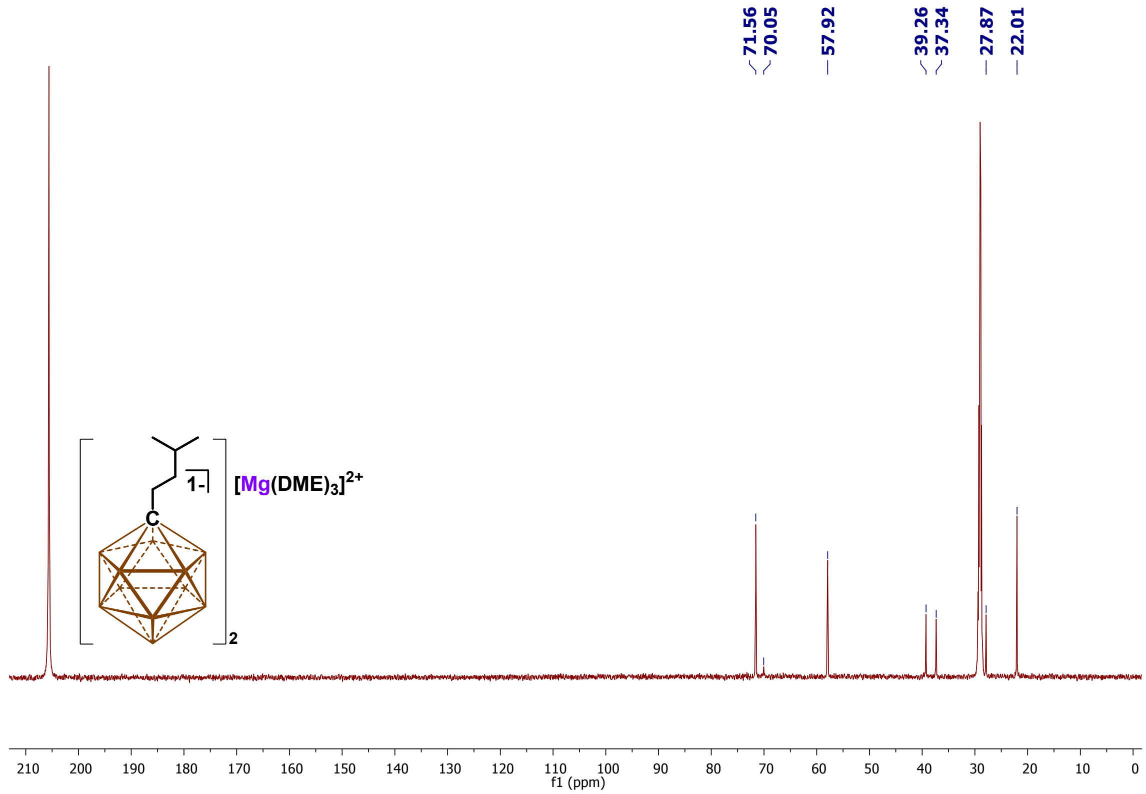

Fig S28. ^{13}C NMR of Mg**2g** in d_6-acetone

Mass Spectrometry

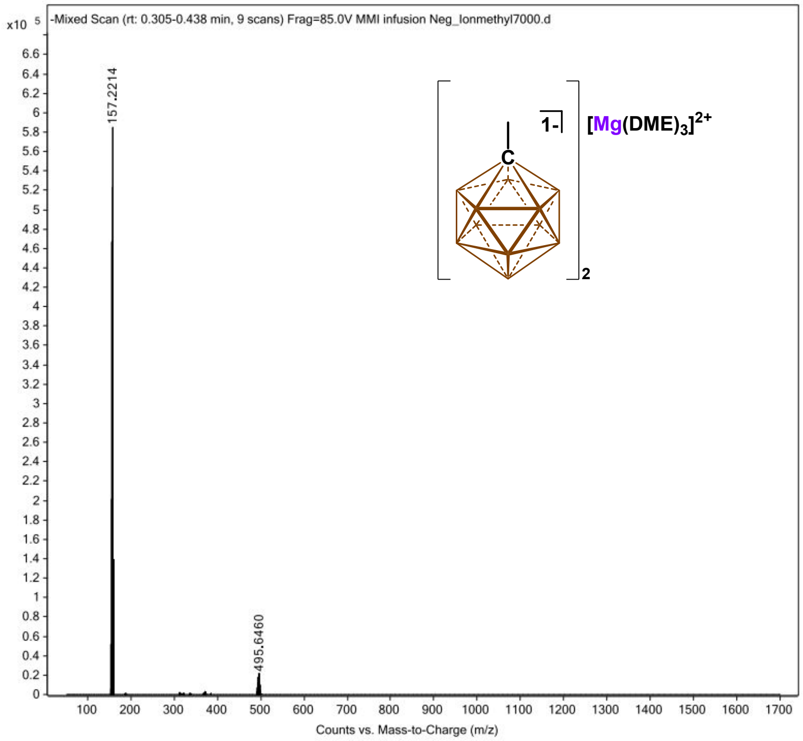

Fig. S29: High Resolution Mass spectrum of Mg**2a**. Note: high mass peak corresponds to a $[Mg(CH_3CB_{11}H_{11})_3]^-$ aggregate generated upon ionization of species.

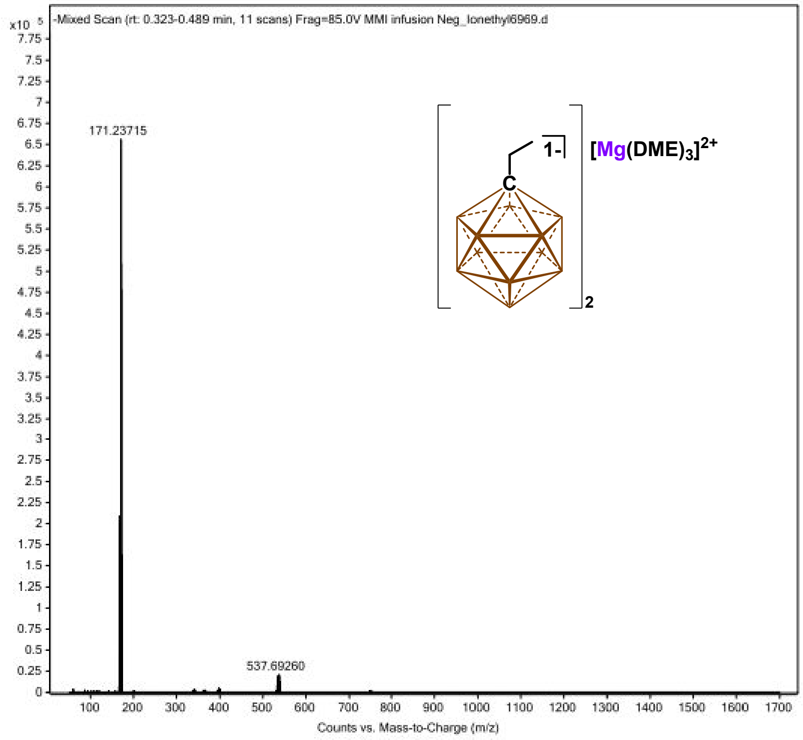

Fig. S30: High Resolution Mass Spectrum of Mg**2b.** Note: high mass peak corresponds to a $[Mg(C_2H_5CB_{11}H_{11})_3]^-$ aggregate generated upon ionization of species.

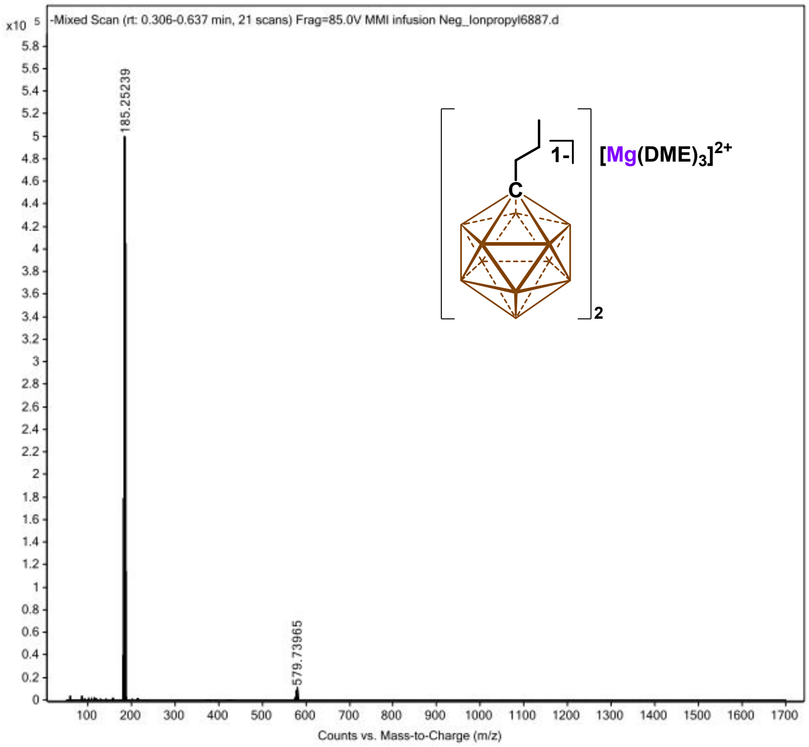

Fig. S31: High Resolution Mass Spectrum of Mg**2c.** Note: high mass peak corresponds to a $[Mg(C_3H_7CB_{11}H_{11})_3]^-$ aggregate generated upon ionization of species.

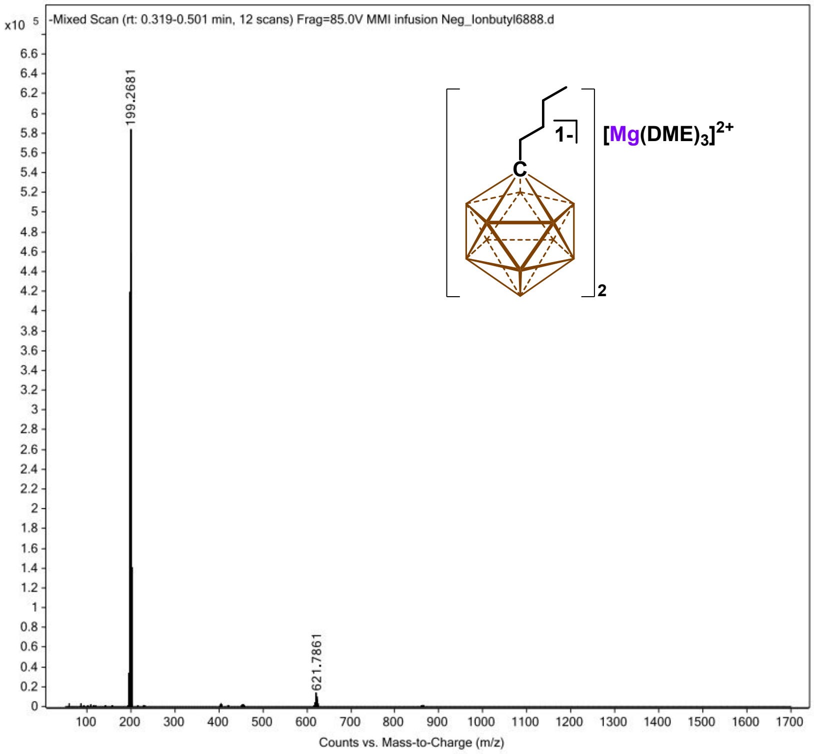

Fig. S32: High Resolution Mass Spectrum of Mg**2d.** Note: high mass peak corresponds to a $[Mg(C_4H_9CB_{11}H_{11})_3]^-$ aggregate generated upon ionization of species.

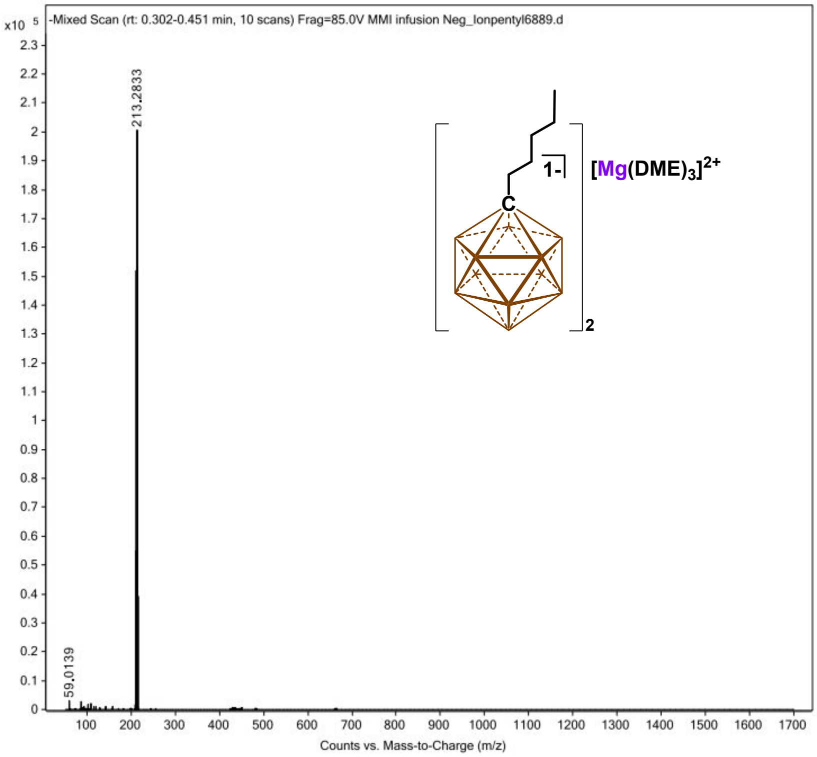

Fig. S33: High Resolution Mass Spectrum of Mg**2e.**

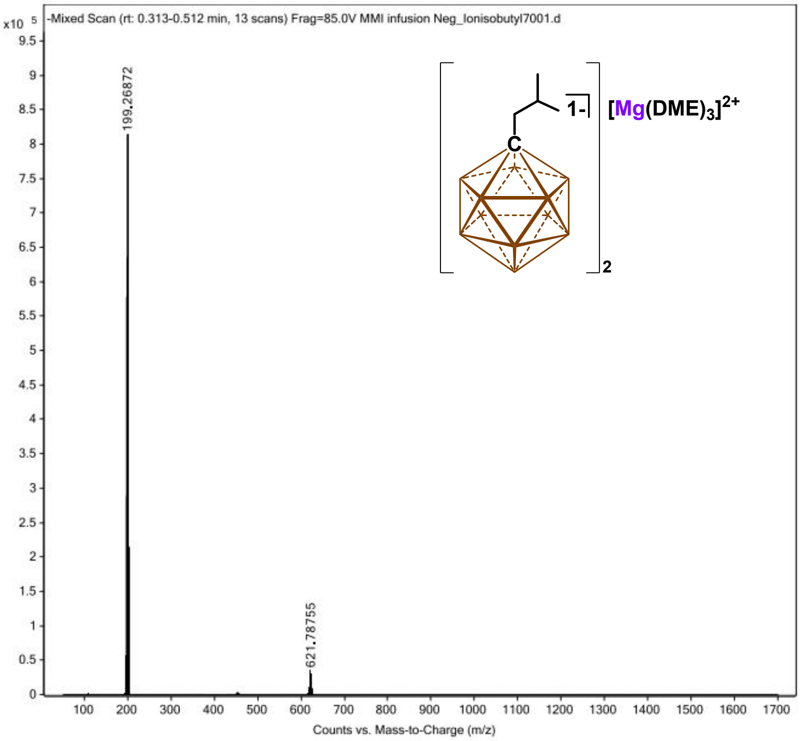

Fig. S34: High Resolution Mass Spectrum of Mg**2f** Note: high mass peak corresponds to a $[Mg(C_4H_9CB_{11}H_{11})_3]^-$ aggregate generated upon ionization of species.

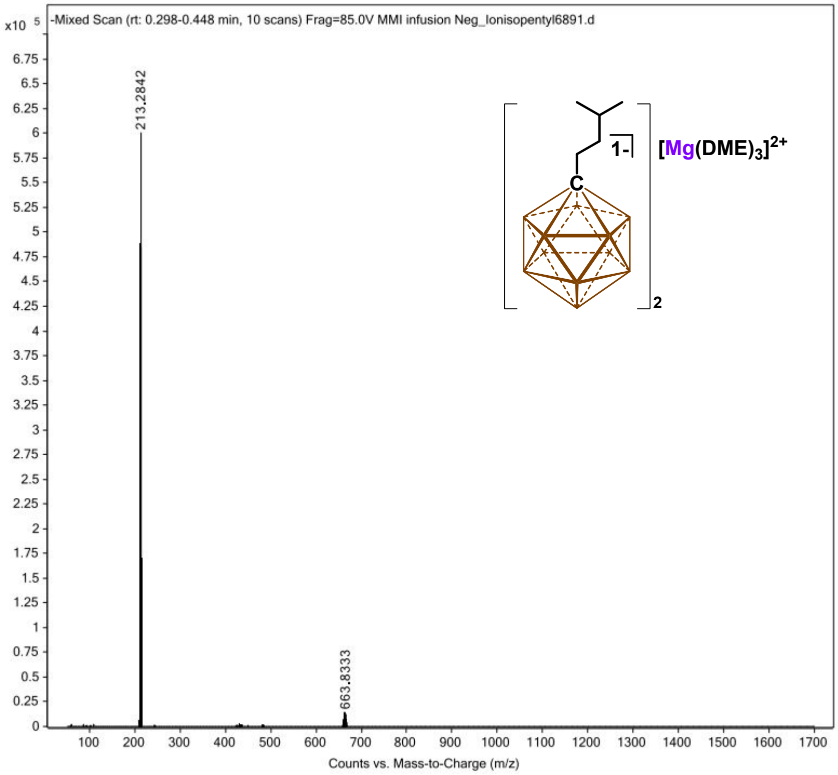

Fig. S35: High Resolution Mass Spectrum of Mg**2g**. Note: high mass peak corresponds to a $[Mg(C_5H_{11}CB_{11}H_{11})_3]^-$ aggregate generated upon ionization of species.

$^{11}B\{^1H\}$ NMR

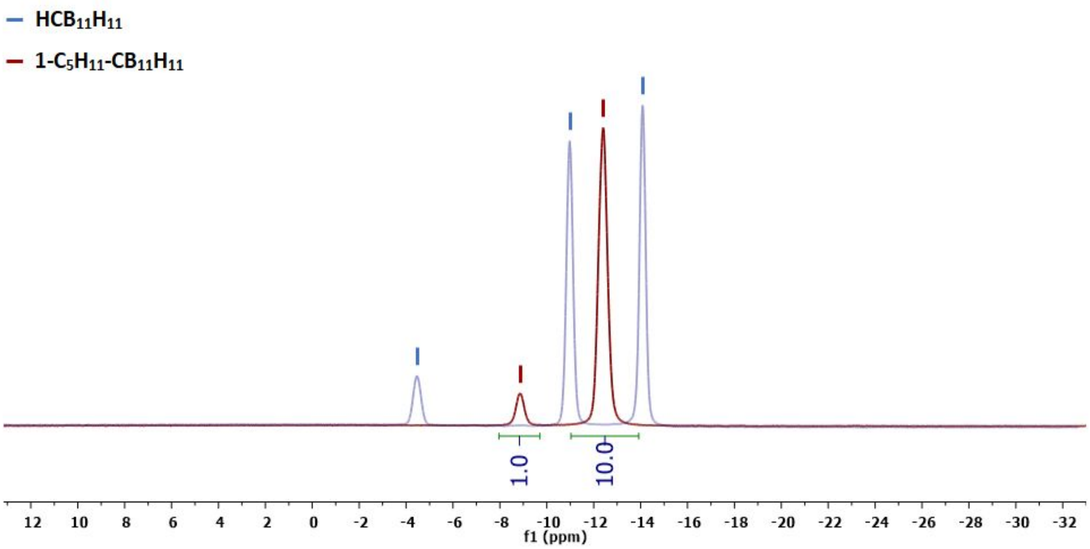

Fig. S36: $^{11}B\{^1H\}$ NMR of alkylated anion **2g** overlayed with unfunctionalized anion **1a**.

Electrolyte Crystallization Behavior

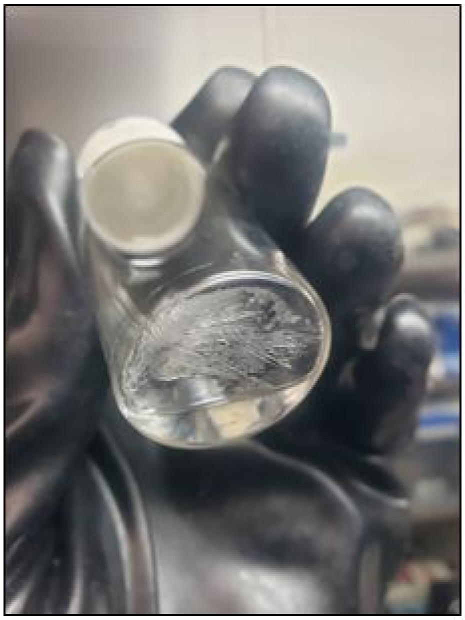

Figure S37: Mg**2a** in DME after heating at 60 °C for 1 hour. In contrast to Mg**1a**, complete dissolution of Mg**2a** was observed after allowing the solution to cool to room temperature.

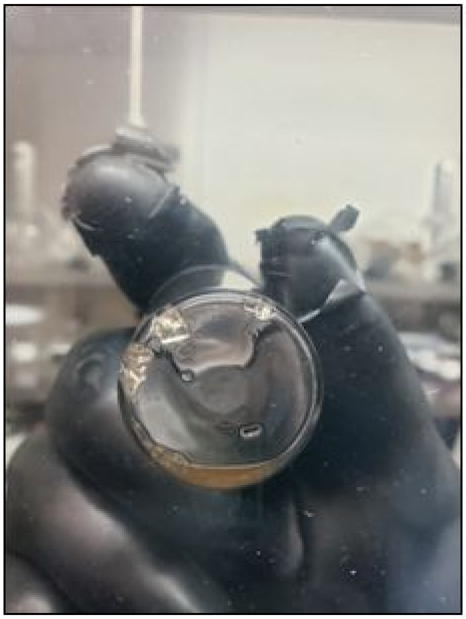

Figure S38: Crystallized Mg**2f** from a concentrated solution in DME at room temperature (1.4 M).

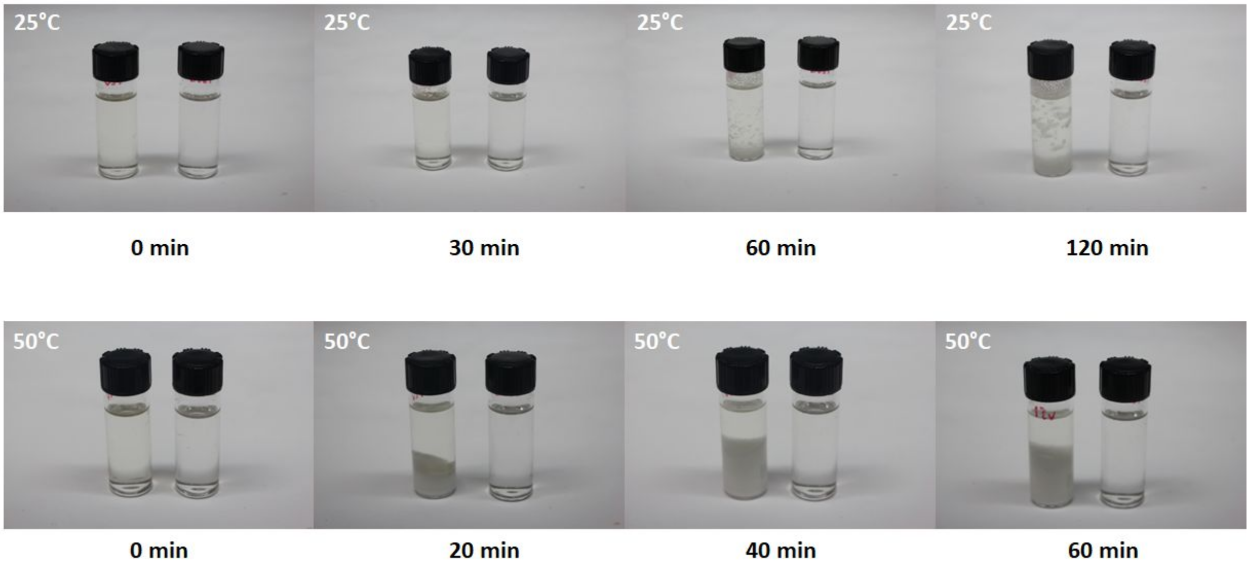

Figure S39: Crystallization behavior of 0.75M Mg**1a** in DME (left) vs. 0.8M Mg**2g** in DME (right) allowed to warm from -30 °C at (a) 25 °C over a period of 2 hours and (b) 50 °C over a period of 1 hour.

Raman Spectroscopy

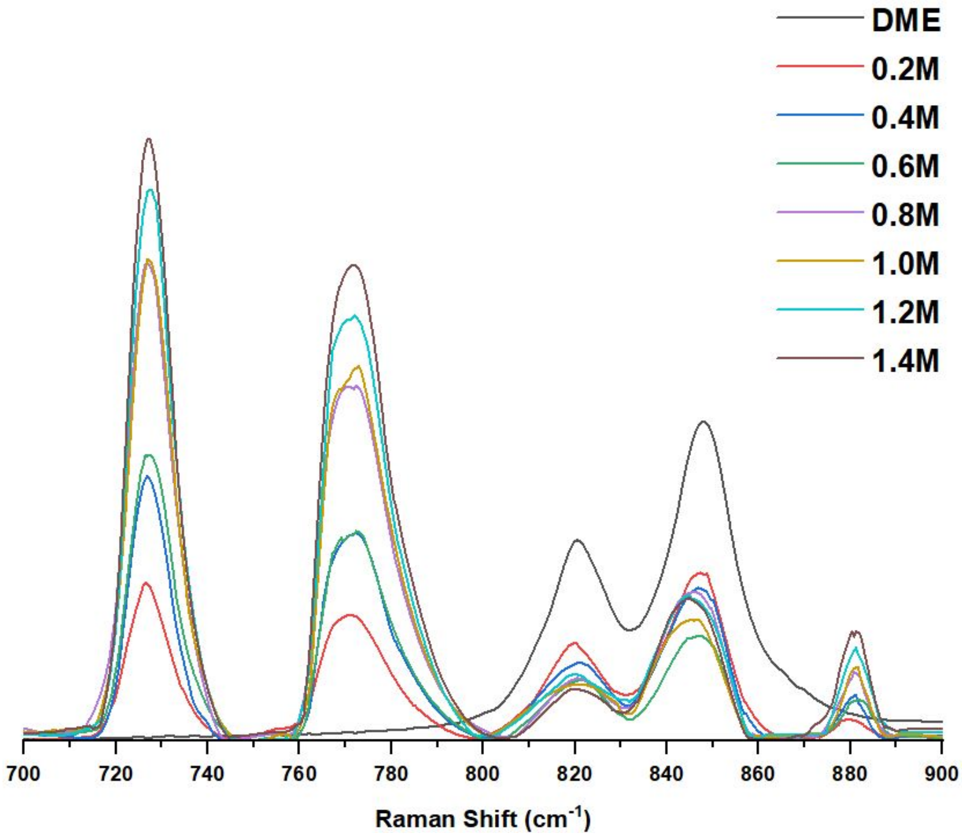

Figure S40: Raman spectroscopy in low frequency region (700 – 900 cm^{-1}) of concentrated DME solutions of Mg**2g**. Depression of peak at 820 cm^{-1} associated with a bending mode of DME is accompanied by gradual increase of peak at 881 cm^{-1} associated with complexation in $[Mg(DME)_3]^{2+}$.

Electrochemistry & Surface Characterization

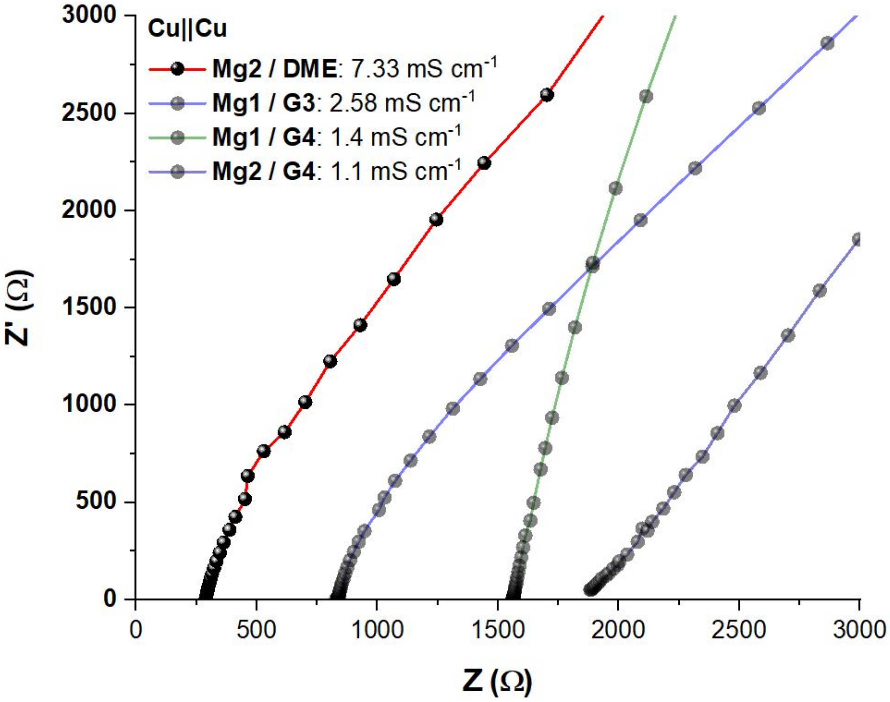

Figure S41: Electrochemical Impedance Spectroscopy of Mg**2g**/DME (red) 0.75M Mg**1a** / G3 (blue) 0.75M Mg**1a** / G4 (navy) 0.8M Mg**2g** / G4.

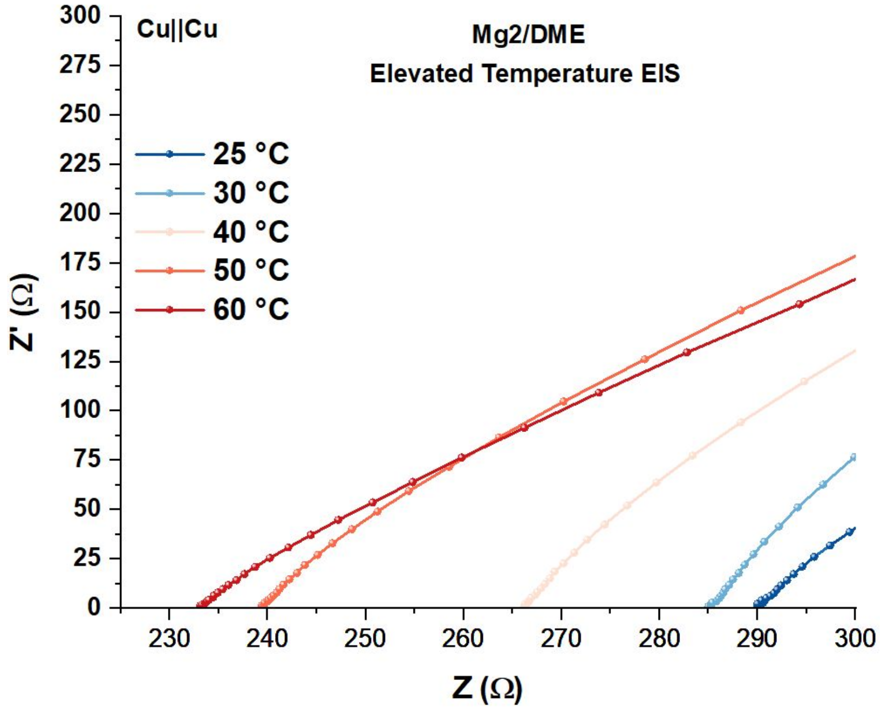

Figure S42: Electrochemical Impedance Spectroscopy of 0.8 M Mg**2g**/DME as a function of temperature.

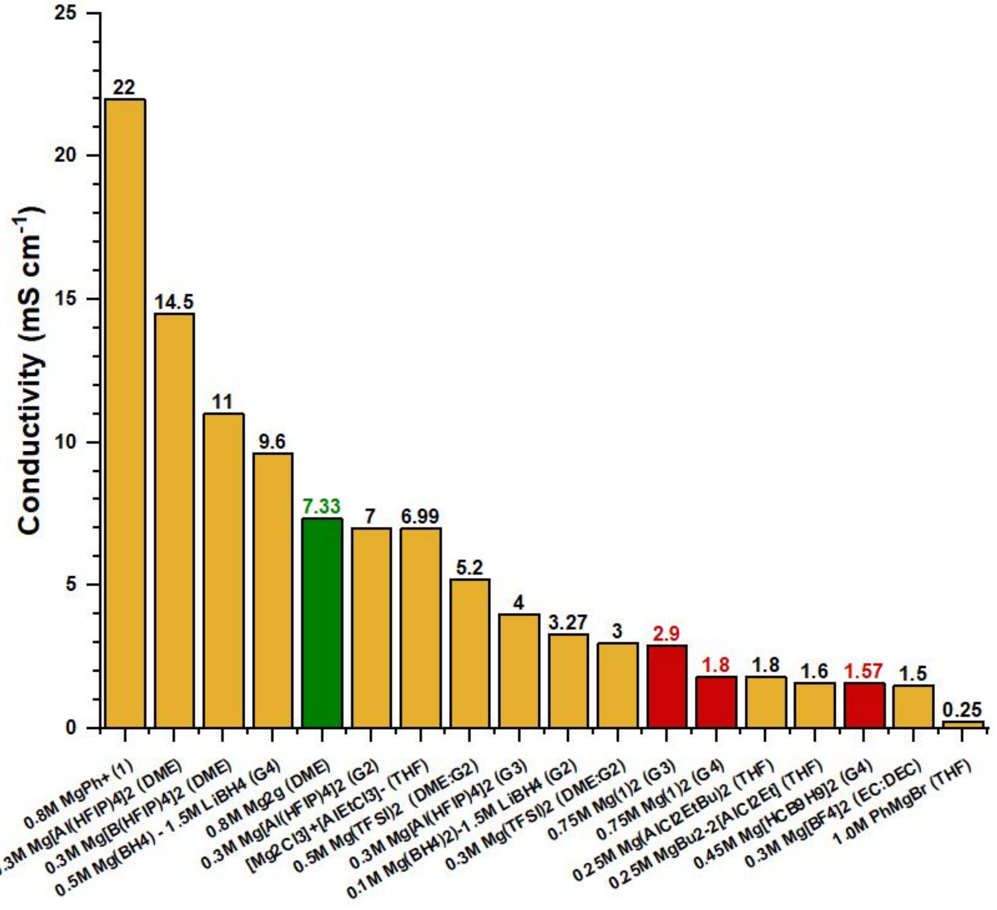

Figure S43: A number of previously reported liquid magnesium electrolytes plotted in order of increasing ionic conductivity including Mg**2g**/DME (green) and previously investigated carboranyl electrolytes (red).

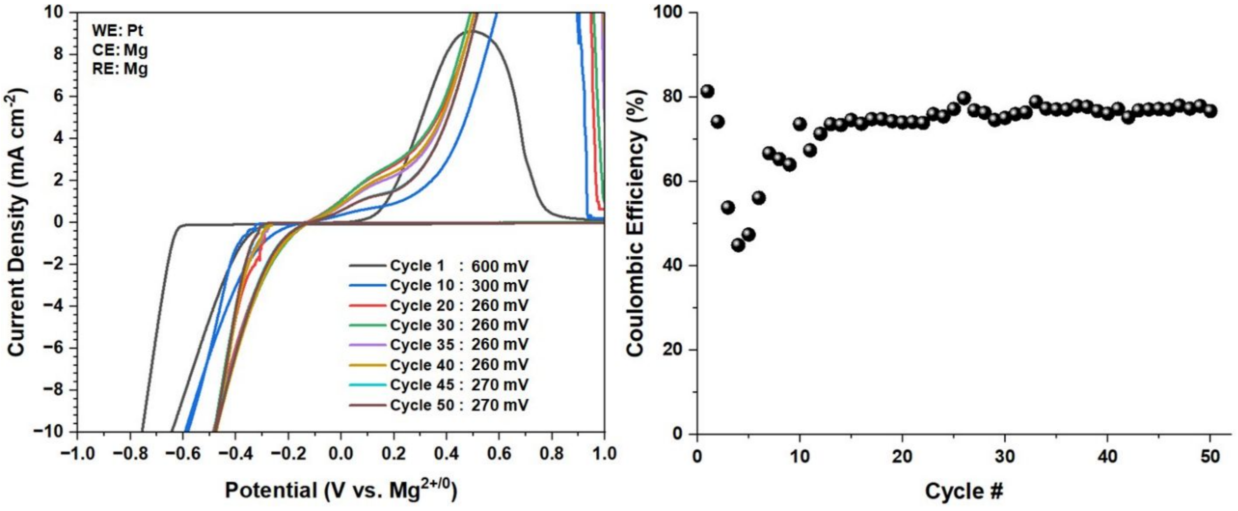

Figure S44: (left) Zoomed in view of Mg**2g** 3-electrode cyclic voltammetry with measured overpotential for displayed cycles (related to Fig. 4d) and (right) corresponding coulombic efficiency over entire 50 cycles.

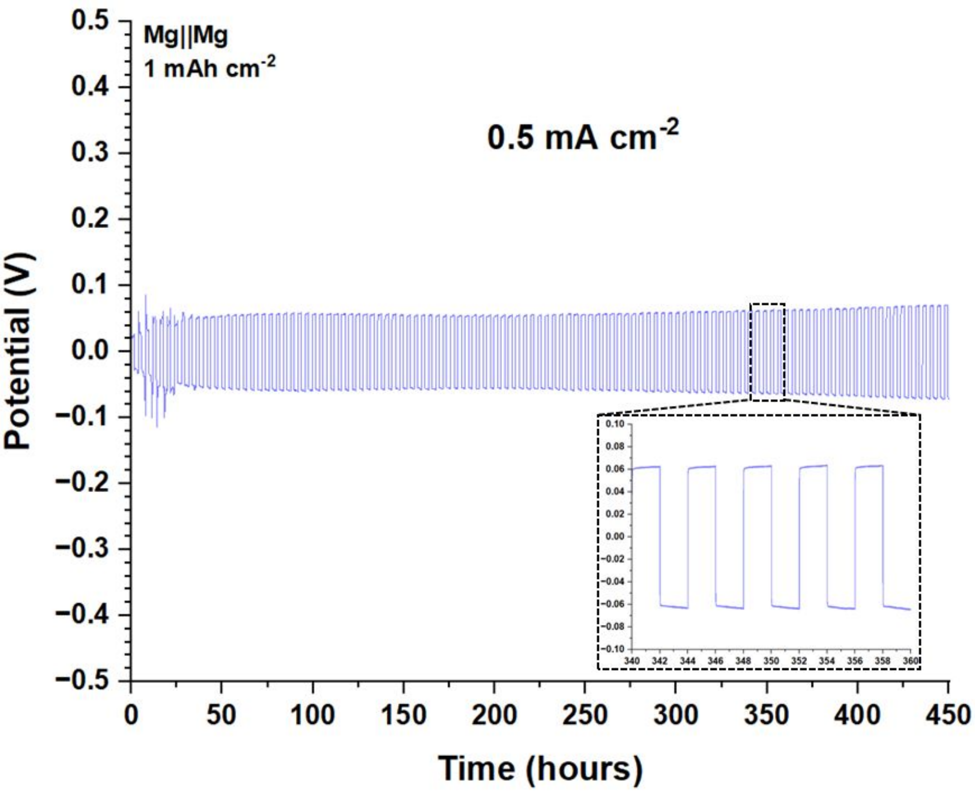

Figure S45: Symmetrical Cell containing Mg**2g**/DME cycled at 0.5 mA cm^{-2}, 1 mAh cm^{-2} areal capacity

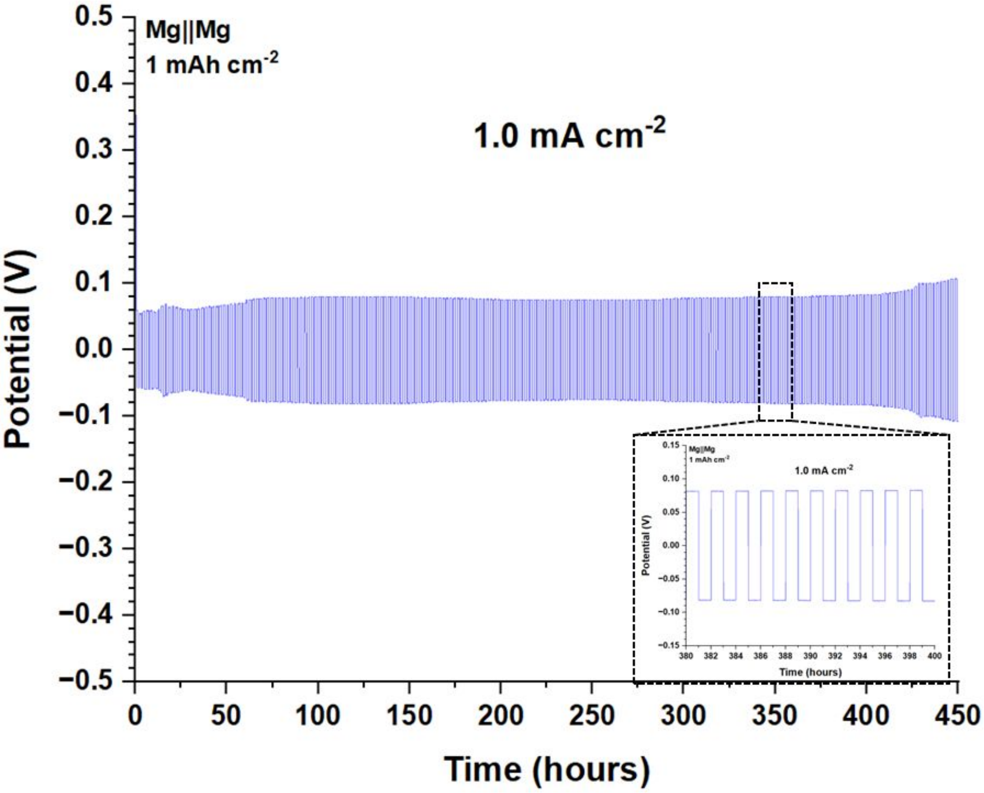

Figure S46: Symmetrical Cell containing Mg**2g**/DME cycled at 1.0 mA cm^{-2}, 1 mAh cm^{-2} areal capacity

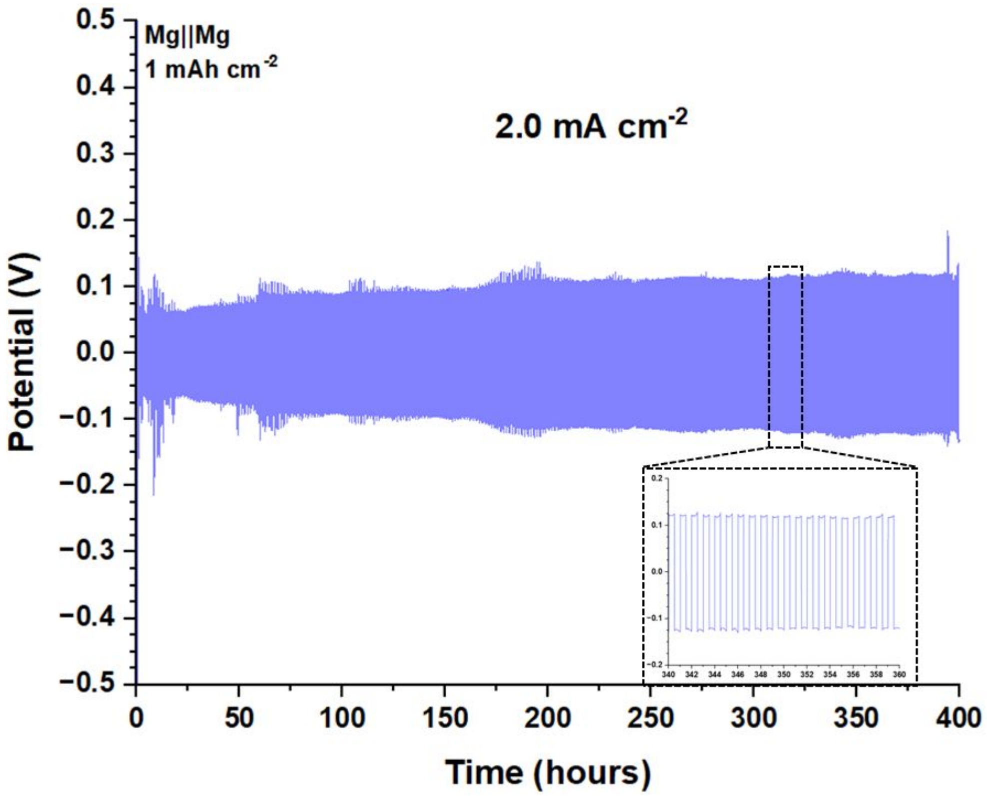

Figure S47: Symmetrical Cell containing Mg**2g**/DME cycled at 2.0 mA cm^{-2}, 1 mAh cm^{-2} areal capacity

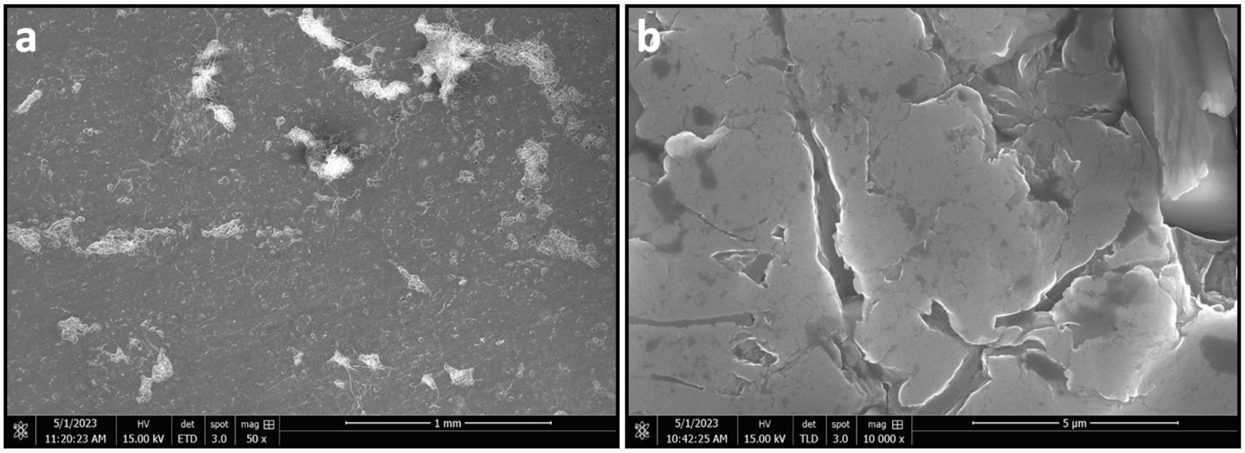

Figure S48: SEM images of magnesium metal deposition on copper (a) 50x magnification of surface and (b) 10kx magnification of surface with Mg**2g**/DME

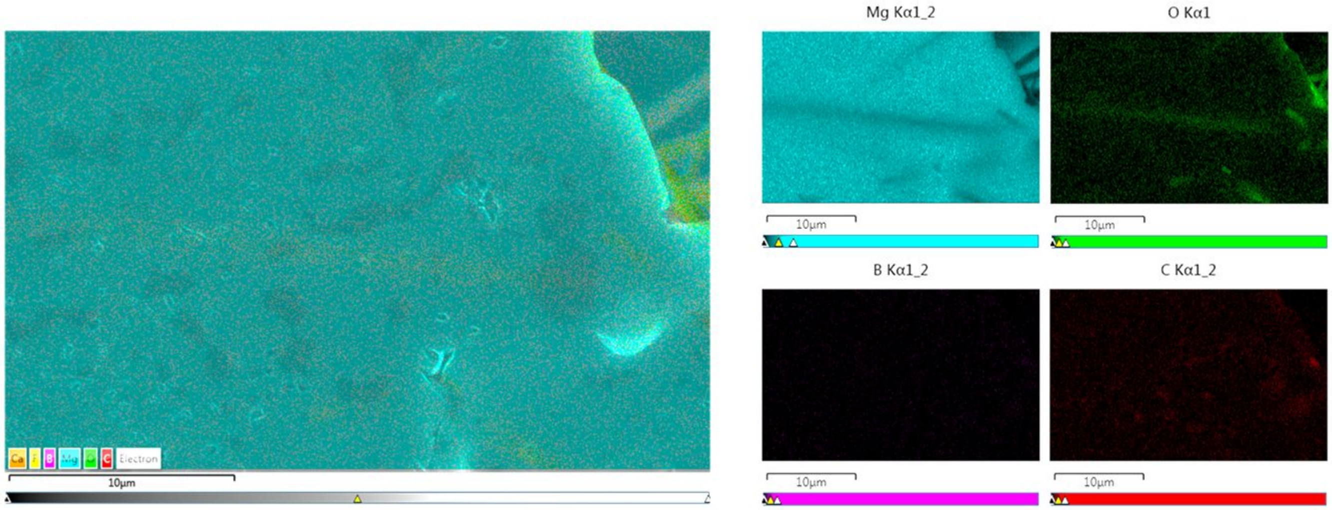

Figure S49: EDS mapping of magnesium metal deposited on copper substrate with Mg**2g**/DME

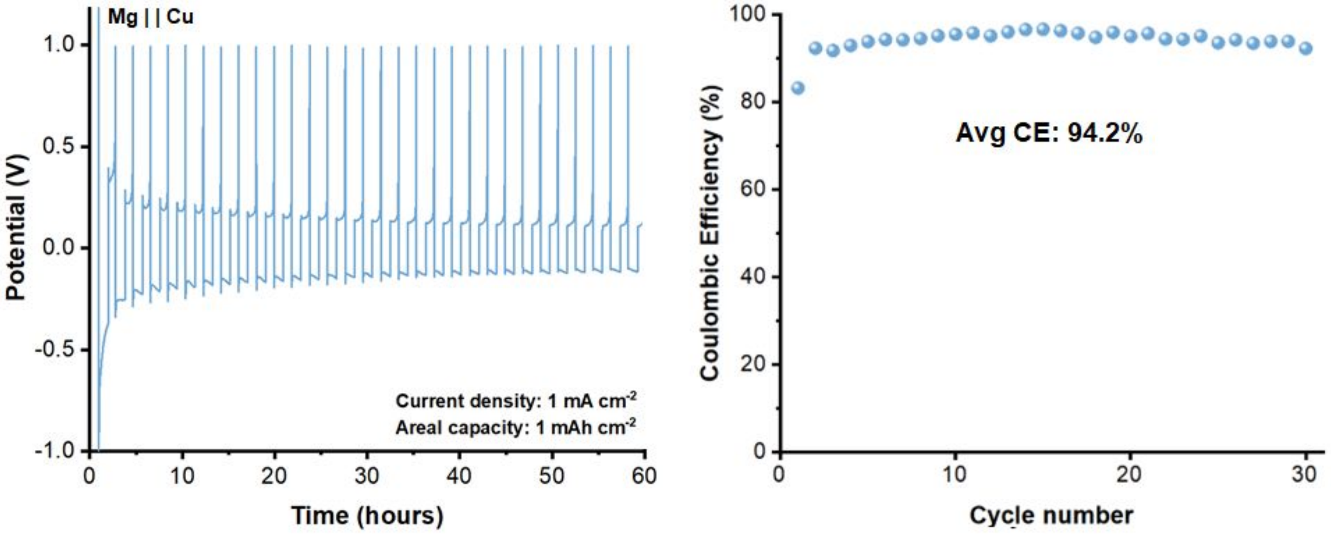

Figure S50: Mg || Cu half cell performance of Mg**2g**/DME (a) voltage profile of cell cycled at 1 mAh cm^{-2} over 60 hours and (b) corresponding coulombic efficiency associated with Mg deposition / stripping processes.

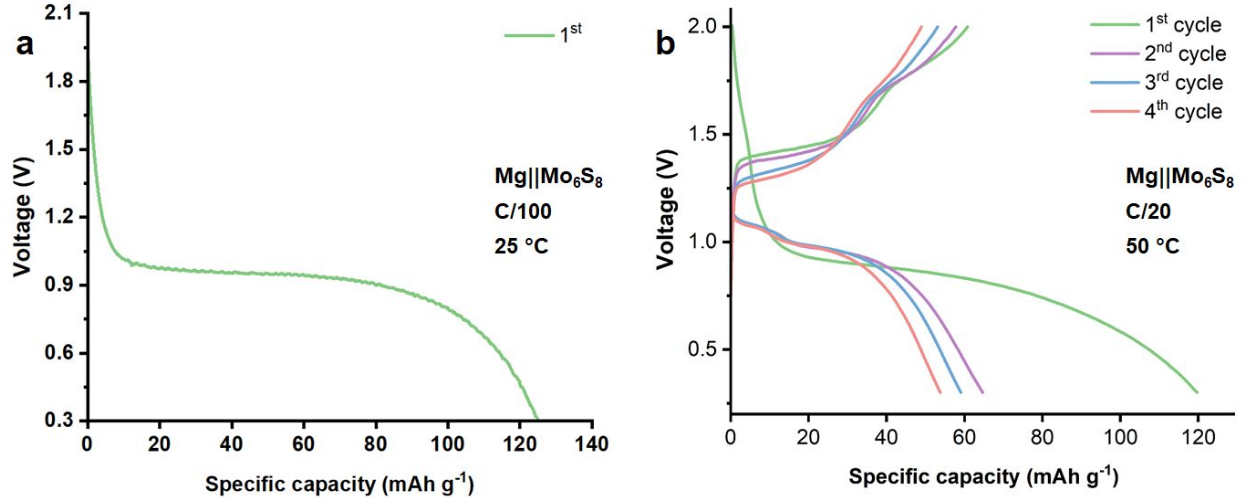

Figure S51: Mg||Mo_6S_8 half cells prepared with Mg**2g**/DME (a) 1st cycle discharge behavior of cell operated at 0.01C, 25 °C (b) an identical cell operated at 0.05C, 50 °C.

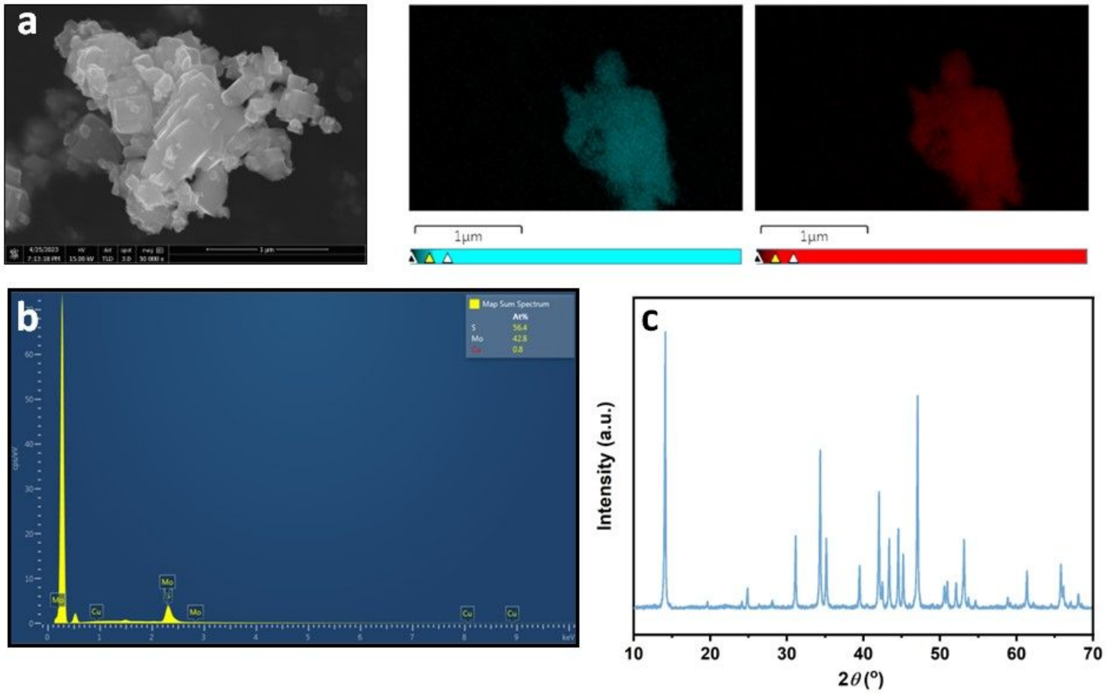

Figure S52: Characterization of synthesized Mo_6S_8 active material (a) SEM and corresponding elemental mapping of Mo_6S_8 (b) EDX spectra of Mo_6S_8 and (c) XRD of Mo_6S_8.

Single Crystal Data – Mg2g

Table S1. Crystal data and structure refinement for Mg2g

Empirical formula	C52.89 H160.67 B44 Mg2 O14.45	
Formula weight	1552.61	
Crystal color, shape, size	colourless plate, 0.49 × 0.16 × 0.12 mm^3	
Temperature	180.00 K	
Wavelength	0.71073 Å	
Crystal system, space group	Orthorhombic, $Pna2_1$	
Unit cell dimensions	a = 37.502(2) Å	α = 90°.
	b = 27.176(2) Å	β = 90°.
	c = 10.7182(8) Å	γ = 90°.
Volume	10923.7(14) $Å^3$	
Z	4	
Density (calculated)	0.944 g/cm^3	
Absorption coefficient	0.066 mm^{-1}	
F(000)	3350	
**Data collection**		
Diffractometer	Bruker D8 Venture	
Theta range for data collection	1.319 to 25.503°.	
Index ranges	-40<=h<=45, -32<=k<=32, -12<=l<=12	
Reflections collected	260458	
Independent reflections	20059 [R_{int} = 0.1394]	
Observed Reflections	13828	
Completeness to theta = 25.242°	99.9 %	

Solution and Refinement

Absorption correction	multi-scan
Max. and min. transmission	0.7452 and 0.6575
Solution	Intrinsic methods
Refinement method	Full-matrix least-squares on F^2
Weighting scheme	$w = [\sigma^2 Fo^2 + AP^2 + BP]^{-1}$, with
	$P = (Fo^2 + 2\ Fc^2)/3$, A = 0.1633, B = 3.2412
Data / restraints / parameters	20059 / 1728 / 1054
Goodness-of-fit on F^2	1.028
Final R indices [$I>2\sigma(I)$]	R1 = 0.0909, wR2 = 0.2302
R indices (all data)	R1 = 0.1291, wR2 = 0.2624
Absolute structure parameter	-0.05(10)
Largest diff. peak and hole	0.691 and -0.428 e.Å^{-3}

References

(1) Reed, C. A. H+, CH3+, and R3Si+ Carborane Reagents: When Triflates Fail. *Accounts of Chemical Research* **2010**, *43* (1), 121-128. DOI: 10.1021/ar900159e.
(2) Jelinek, T.; Baldwin, P.; Scheidt, W. R.; Reed, C. A. New weakly coordinating anions. 2. Derivatization of the carborane anion CB11H12. *Inorganic Chemistry* **1993**, *32* (10), 1982-1990. DOI: 10.1021/ic00062a018.
(3) Geng, L.; Lv, G.; Xing, X.; Guo, J. Reversible Electrochemical Intercalation of Aluminum in Mo6S8. *Chemistry of Materials* **2015**, *27* (14), 4926-4929. DOI: 10.1021/acs.chemmater.5b01918.
(4) SAINT, V. A., Bruker Analytical X-Ray Systems, Madison, WI. **2012**.
(5) SADABS, Bruker Analytical X-Ray Systems, Madison, WI. **2016**.
(6) Sheldrick, G. SHELXT - Integrated space-group and crystal-structure determination. *Acta Crystallographica Section A* **2015**, *71* (1), 3-8.
(7) Sheldrick, G. M. *Acta Cryst. A64* **2008**, 112-122.
(8) Dolomanov, O. V. *J. Appl. Crystallogr.* **2009**, *42*, 339-341.
